# Mycobacterium leprae diversity and population dynamics in medieval Europe from novel ancient genomes

**DOI:** 10.1186/s12915-021-01120-2

**Published:** 2021-10-05

**Authors:** Saskia Pfrengle, Judith Neukamm, Meriam Guellil, Marcel Keller, Martyna Molak, Charlotte Avanzi, Alena Kushniarevich, Núria Montes, Gunnar U. Neumann, Ella Reiter, Rezeda I. Tukhbatova, Nataliya Y. Berezina, Alexandra P. Buzhilova, Dmitry S. Korobov, Stian Suppersberger Hamre, Vitor M. J. Matos, Maria T. Ferreira, Laura González-Garrido, Sofia N. Wasterlain, Célia Lopes, Ana Luisa Santos, Nathalie Antunes-Ferreira, Vitória Duarte, Ana Maria Silva, Linda Melo, Natasa Sarkic, Lehti Saag, Kristiina Tambets, Philippe Busso, Stewart T. Cole, Alexei Avlasovich, Charlotte A. Roberts, Alison Sheridan, Craig Cessford, John Robb, Johannes Krause, Christiana L. Scheib, Sarah A. Inskip, Verena J. Schuenemann

**Affiliations:** 1 Institute of Evolutionary Medicine, University of Zurich, Winterthurerstrasse 190, 8057 Zurich, Switzerland; 2 Institute for Archaeological Sciences, University of Tübingen, Rümelinstrasse 19-23, 72070 Tübingen, Germany; 3 Institute for Bioinformatics and Medical Informatics, University of Tübingen, Sand 14, 72076 Tübingen, Germany; 4 Estonian Biocentre, Institute of Genomics, University of Tartu, Riia 23B, 51010 Tartu, Estonia; 5 Centre of New Technologies, University of Warsaw, S. Banacha 2c, 02-097 Warsaw, Poland; 6 Mycobacteria Research Laboratories, Department of Microbiology, Immunology and Pathology, Colorado State University, Fort Collins, USA; 7 Swiss and Tropical Public Health Institute, Basel, Switzerland; 8 Unitat d’Antropologia Biològica, Departament de Biologia Animal, Biologia Vegetal i Ecologia, Universitat Autònoma de Barcelona, 08193 Bellaterra (Cerdanyola del Vallès), Barcelona, Spain; 9 Max Planck Institute for the Science of Human History, Kahlaische Str. 10, 07745 Jena, Germany; 10 Laboratory of Structural Biology, Kazan Federal University, Kazan, Russian Federation 420008; 11 Research Institute and Museum of Anthropology, Moscow State University, 125009, Mokhovaya str. 11, Moscow, Russian Federation; 12 The Institute of Archaeology of the Russian Academy of Sciences, 117292, Dm. Uljanova str. 19, Moscow, Russian Federation; 13 Department of Archaeology, History, Cultural studies and religion, University of Bergen, 5020 Bergen, Norway; 14 Department of Life Sciences, University of Coimbra, Research Centre for Anthropology and Health, Calçada Martim de Freitas, 3000-456 Coimbra, Portugal; 15 Laboratory of Forensic Anthropology, Department of Life Sciences, University of Coimbra, Centre for Functional Ecology, Calçada Martim de Freitas, 3000-456 Coimbra, Portugal; 16 Área de Antropología Física, Departamento de Biodiversidad y Gestión Ambiental, Universidad de León, Campus de Vegazana, 24071 León, Spain; 17 Institute of Biomedicine (IBIOMED), Universidad de León, Campus de Vegazana, 24071 León, Spain; 18 Laboratory of Biological Anthropology, Department of Biology; School of Science and Technology, University of Évora, Évora, Portugal; 19 Laboratório de Ciências Forenses e Psicológicas Egas Moniz (LCFPEM), Centro de Investigação Interdisciplinar Egas Moniz (CiiEM), Instituto Universitário Egas Moniz, Egas Moniz CRL, Monte de Caparica, Portugal; 20 Laboratory of Biological Anthropology and Human Osteology (LABOH), CRIA/FCSH, Universidade NOVA de Lisboa, Lisbon, Portugal; 21 UNIARQ – University of Lisbon, Lisbon, Portugal; 22 OSTEO Research, Camino de la Iglesia 1, Barrio de mata, Santiuste De Pedraza, 40171 Segovia, Spain; 23 Global Health Institute, Ecole Polytechnique Fédérale de Lausanne, Lausanne, Switzerland; 24 Institut Pasteur, 25-28, rue du Docteur Roux, 75724 Paris Cedex 15, France; 25 Department of Archeology, History of Belarus and Special Historical Disciplines, Mogilev State A. Kuleshov University, Str Kosmonavtov 1, Mogilev, 212022 Republic of Belarus; 26 Department of Archaeology, Durham University, South Road, Durham, DH1 3 LE UK; 27 Department of Scottish History and Archaeology, National Museums Scotland, Chambers Street, Edinburgh, EH1 1JF UK; 28 Department of Archaeology, University of Cambridge, Downing Street, Cambridge, CB2 3ER UK; 29 Senckenberg Centre for Human Evolution and Paleoenvironments, University of Tübingen, Rümelinstrasse 19-23, 72070 Tübingen, Germany; 30 St John’s College, University of Cambridge, Cambridge, CB2 1TP UK; 31 School of Archaeology and Ancient History, University of Leicester, Leicester, LE1 7RH UK

**Keywords:** Ancient DNA, Ancient pathogen genomics, *Mycobacterium leprae*, Pathogen diversity, Leprosaria, Pathogen population dynamics, Paleomicrobiology, Paleopathology

## Abstract

**Background:**

Hansen’s disease (leprosy), widespread in medieval Europe, is today mainly prevalent in tropical and subtropical regions with around 200,000 new cases reported annually. Despite its long history and appearance in historical records, its origins and past dissemination patterns are still widely unknown. Applying ancient DNA approaches to its major causative agent, *Mycobacterium leprae*, can significantly improve our understanding of the disease’s complex history. Previous studies have identified a high genetic continuity of the pathogen over the last 1500 years and the existence of at least four *M. leprae* lineages in some parts of Europe since the Early Medieval period.

**Results:**

Here, we reconstructed 19 ancient *M. leprae* genomes to further investigate *M. leprae’s* genetic variation in Europe, with a dedicated focus on bacterial genomes from previously unstudied regions (Belarus, Iberia, Russia, Scotland), from multiple sites in a single region (Cambridgeshire, England), and from two Iberian leprosaria. Overall, our data confirm the existence of similar phylogeographic patterns across Europe, including high diversity in leprosaria. Further, we identified a new genotype in Belarus. By doubling the number of complete ancient *M. leprae* genomes, our results improve our knowledge of the past phylogeography of *M. leprae* and reveal a particularly high *M. leprae* diversity in European medieval leprosaria.

**Conclusions:**

Our findings allow us to detect similar patterns of strain diversity across Europe with branch 3 as the most common branch and the leprosaria as centers for high diversity. The higher resolution of our phylogeny tree also refined our understanding of the interspecies transfer between red squirrels and humans pointing to a late antique/early medieval transmission. Furthermore, with our new estimates on the past population diversity of *M. leprae*, we gained first insights into the disease’s global history in relation to major historic events such as the Roman expansion or the beginning of the regular transatlantic long distance trade. In summary, our findings highlight how studying ancient *M. leprae* genomes worldwide improves our understanding of leprosy’s global history and can contribute to current models of *M. leprae*’s worldwide dissemination, including interspecies transmissions.

**Supplementary Information:**

The online version contains supplementary material available at 10.1186/s12915-021-01120-2.

## Background

Hansen’s disease (leprosy), caused by infection with *Mycobacterium leprae* or *Mycobacterium lepromatosis*, is one of the oldest recorded diseases known to humankind. Its notoriety relates both to its potential to cause extreme physical manifestations of infection, which include damage to the peripheral nervous system, mucosal membranes, skin, and ultimately the extremities [1], and its misattribution to a disease in biblical texts [2, 3]. The earliest probable descriptions of the disease are from Egyptian papyri from 1550 BCE and the Sushruta Samhita (600 BCE) from India [4]. More reliable accounts of Hansen’s disease are found in ancient Greek and Roman literature [5] from the first century CE onwards [6]. This geographically focused information led some historians to suggest that the disease may have originated in Africa [7], although most agree on a likely origin in Asia, possibly in the region of today’s India [8]. It was thought to have travelled west during the conquests of Alexander the Great (fourth century BCE) or through trading and likely then diffused around the Mediterranean basin and into Western Europe with the expansion of the Roman Empire (200 BCE–600 CE) [9].

Geospatial analyses of archeological skeletons of individuals with Hansen’s disease have done little to change this narrative. Until recently, the earliest individuals with evidence for infection have been dated to the 2nd millennium BCE in India [8], fourth to third centuries BCE in Italy [10], third century BCE in Egypt [11], and first century CE in Israel [12, 13]. However, the recent identification of two possible, albeit genetically unconfirmed cases from Bronze Age Hungary (4th millennium BCE) [14] and Early Bronze Age Scotland (late 3rd millennium to early 2nd millennium BCE) [1, 15] throws a simple eastern Asian origin hypothesis into question. In addition, there is evidence from 3rd to 2nd millennium BCE Pakistan [16] as well as possible evidences from Nubia, 2300 BCE [17], Iran, 6200-5700 cal BCE [18], and Turkey dated to 2300 BCE [19], that all need further investigation.

Archeological and historical sources all demonstrate that Hansen’s disease was widespread in Europe by the Middle Ages, being increasingly identified from the Roman and early Medieval periods (200 BCE–600 CE). The period from 1000 to 1300 CE saw the increased foundation of leprosaria across the continent [20]. These charitable institutions, often running under monastic rules, were set up to receive and support individuals who had “leprosy,” which not only included people with Hansen’s disease, but likely encompassed those with other conditions that medieval people also diagnosed as “leprosy” [21, 22]. They also received individuals with other diagnosed diseases, increasingly so in the late medieval period [21, 22]. Despite popular belief, people were not forced to live in these institutions when diagnosed, and could even be expelled, but they often paid for their residence and would receive spiritual and practical support [23]. Modern excavations of leprosarium cemeteries show tens to several hundreds of people buried in them often without skeletal evidence of Hansen’s disease [24–28]. Of the archeological cemeteries not related to a leprosarium where Hansen’s disease has been identified in skeletons, the majority show that infected people were buried in the common manner for their location and time period [1]. For medieval Europe, this includes, for example, Norwich, England [29]; Kirk Hill, Scotland [30, 31]; multiple sites in Schleswig, Denmark/Germany [32]; Seville [33] and Gijon in Spain [34]; Beja, Portugal [35], and Kaldus, Poland [36]—see also [1] for a global view.

For as yet unclear reasons, the disease prevalence began to decline in Europe from the fourteenth century, although pockets of infection remained until the nineteenth century, e.g., in Scandinavia [1] or even until the twentieth century in Spain [37]. Currently accepted hypotheses for its decline include cross-immunity offered by tuberculosis infection [38], or a loss of susceptible hosts due to the rise of other competing infectious diseases (plague, tuberculosis, etc.), or changes in hygienic practices including the construction of leprosaria [26]. For an overview of potential causes for the decline, see [1], and references within. While autochthonous cases are rarely reported in Europe today, the disease remains a significant social challenge in Brazil, India, and Indonesia with up to 200,000 new cases globally per year [39]. In 2020, the World Health Organization cites 177,175 “registered cases” and 202,185 “new cases” for the end of 2019, of which 71% were reported from South and East Asia [40].

The understanding of *M. leprae*’s evolutionary history benefits from the genetic investigation of archeological human remains (skeletons or preserved bodies such as mummies). Initially, PCR-based analyses identified four major SNP (single-nucleotide polymorphism) types from 1 to 4, which allowed an assessment of the phylogeographic distribution of archeological evidence within a framework of modern strain distribution [41]. Later, the four major SNP types were resolved into 16 subgroups from A to P [42]. At this point of studying modern and ancient *M. leprae*, the distribution of SNP types correlated with geographic location and could largely be explained by major population movements [42, 43]. Prior to the introduction of the branch system and the SNP subtyping, it was argued that while the ancestral SNP type 2 strains originated in Africa, Hansen’s disease spread westwards giving rise to SNP type 3 and eastwards resulting in SNP type 1 [42]. Further, it was suggested that SNP type 4 emerged in West Africa [42]. Due to technical improvements in the field of ancient DNA research, including next-generation sequencing and targeted DNA enrichment methods, the first complete genomes of *M. leprae* from archeological human samples were reconstructed in 2013 [44]. Investigation of the phylogenetic resolution of the ancient genomes initially clustered *M. leprae* genomes into five major branches (0-4) [44]. Later studies including modern and ancient *M. leprae* genomes [45, 46] improved the resolution of branch 2, which was split into branches 2E, 2H, 2F, identified a completely new fifth branch, further named branch 5 as well as correlating SNP subtyping and branching system [45–47]. Both classifications are now used based on the resolution needed for phylogenetic and transmission in ancient and modern *M. leprae* studies [46].

Genome-wide analyses of ancient and modern *M. leprae* DNA strains made it clear that there is no longer a simple correlation between the diverse bacterial genomes and their geographic origins. Hence, questions concerning the origin of leprosy are still unresolved. Ancient DNA studies have uncovered a high genetic diversity of *M. leprae* strains in medieval Europe [44, 45], revealing the possibility that some *M. leprae* strains had spread worldwide from the European continent. For example, it is assumed that Europeans spread branch 3 genomes to the Americas in the sixteenth century and later, through contact via the slave trade [48], where they still exclusively persist [44]. More intriguingly, the ten previously reconstructed ancient genomes demonstrated that nearly all major branches of *M. leprae* seen today, including the most basal (branch 0) currently associated with modern East Asian samples [45, 47], were present in medieval Europe. Furthermore, lineages from three different branches were identified in people buried in the same medieval cemetery at St Jørgen (Odense, Denmark) [45]. Unfortunately, the low number of sequenced genomes from medieval Europe, including from leprosaria, make it difficult to assess how widespread such high local diversity was, or whether it was unique to this particular site. Uncovering such high diversity raises important questions about whether the origin of the disease may in fact be in Eurasia, but the lack of ancient data from outside Europe and the resulting potential sampling bias do not yet allow precise conclusions [45]. Even the recently published oldest genome from an ancient Egyptian mummy from Abusir el-Meleq, thereby representing the only ancient *M. leprae* genome outside Europe, does not further refine the localization of leprosy’s origin [49]. Furthermore, the identification of *M. leprae* in modern red squirrels in Britain [50], genetically closely related to a branch 3 strain isolated in a fifth–sixth century male from the Essex/Cambridgeshire border, eastern England, highlights the possibility that there may be important animal reservoirs that could add a further layer of complexity to the identified diversity [51]. In addition, there are still large parts of Europe for which there is ample historical and archeological evidence for the disease, but for which we have little to no information on the genetic variation of the strains present there.

Here, we address these gaps by examining 41 individuals including 39 with osteological or historical contextual evidence of Hansen’s disease, and two were exclusively identified as positive for leprosy by genetic examination. These individuals originated from 20 archeological sites across Europe dating from the sixth to the twentieth centuries CE including areas for which previously no genome-wide data existed: Belarus, Iberia, Russia, and Scotland. To further assess intra-regional strain diversity, we investigated skeletons from multiple sites of the same region (Cambridgeshire, England) and the remains of people buried in two leprosaria in Portugal and Spain. We were able to reconstruct 19 *M. leprae* genomes which were also suitable for phylogenetic analysis, doubling the number of published ancient genomes, and to identify a new SNP subtype, named 3Q, in Belarus. The analysis of these new genomes supports the previous findings of high genetic diversity in medieval leprosy strains across Europe and indicates that this was also the case in at least one other medieval leprosarium site, the Hospital of Sant Llàtzer (Barcelona).

## Results

### Sample information and dating

Samples from a total of 41 individuals were investigated in this study (Additional file 1: Supplementary Note 1, Table S1 and S2) [1, 21, 30, 31, 35, 50, 52–88]. All but two individuals (JDS097 and BEL024) were previously associated with Hansen’s disease due to either their archeological context or pathological lesions compatible with a diagnosis of Hansen’s disease (Additional file 1: Supplementary Note 1) [1, 21, 30, 31, 35, 50, 52–88], and archeologically dated from the Early Medieval period to the twentieth century (Table 1). Fifteen individuals were radiocarbon dated (Table 1, Additional file 1: Supplementary Note 2, Fig. S1, Table S1) [89–95], seven to the late medieval period (eleventh to fourteenth century), and two to the modern era (fifteenth to twentieth century). The Edix Hill skeleton (Cambridgeshire, eastern England), the earliest investigated here, is archeologically dated to the sixth–seventh centuries, confirmed by radiocarbon dating.

**Table 1:** Overview of all newly sequenced *Mycobacterium leprae* genomes. The age of the samples is either given in archeological dates (italic) or radiocarbon dates. The age of all directly dated samples is provided in calibrated CE. The listed SNP types are determined according to the new SNP typing system [46]. Following this new system, a new SNP was determined for the BEL024 sample (labeled with an asterisk), but according to the SNP typing system by Monot and colleagues [42] the sample would be classified as 3L

Sample	Sample age (^**14**^C dates: non-italics; archeological ages: italics)	Location	Mean coverage	Coverage ≥ 1× in %	Coverage ≥ 3× in %	Genotype (new)	Branch
R7546-671	19th–20th century CE	St. Petersburg, Russia	16.51	97.16	94.96	2F	2F
UF11	*18th century CE*	Sant Llàtzer, Barcelona, Spain	6.71	85.67	61.80	3I-1	3
UF8	*16th century CE*	Sant Llàtzer, Barcelona, Spain	1.46	67.52	18.99	3I-1	3
UF21	1431–1611 cal CE	Sant Llàtzer, Barcelona, Spain	4.11	92.14	67.70	3I-1	3
UF25	1423–1466 cal CE	Sant Llàtzer, Barcelona, Spain	33.09	97.40	95.73	3K-0	0
JDS097	1231–1384 cal CE	Hospital of St. John, Cambridge, UK	12.81	96.89	94.27	3I-1	3
PAVd’09_I.5	1283–1396 cal CE	Valle da Gafaria, Lagos, Portugal	96.82	97.40	97.44	3I-1	3
Bergen	1268–1388 cal CE	Nonneseter, Bergen, Norway	110.61	97.45	97.44	3I-1	3
UF700	1035–1165 cal CE	Sant Llàtzer, Barcelona, Spain	19.45	97.53	96.91	3I-1	3
UF101	1027–1157 cal CE	Sant Llàtzer, Barcelona, Spain	21.28	13.70	97.39	3I-1	3
UF800	*12th*–*early 13th century CE*	Sant Llàtzer, Barcelona, Spain	3.34	86.27	52.63	2F	2F
COR_XVIII	*12th*–*early 13th* *century CE*	Cordiñanes de Valdeón, León, Spain	2.49	67.28	32.39	3K-0	0
UF703	1040–1208 cal CE	Sant Llàtzer, Barcelona, Spain	26.94	97.44	96.19	3K-0	0
KirkHill	1030–1155 cal CE	Kirk Hill, St Andrews, Scotland	6.86	94.85	81.01	3I-1	3
UF803	1023–1157 cal CE	Sant Llàtzer, Barcelona, Spain	6.18	91.01	69.77	3K-0	0
CHRY044	1034–1175 cal CE	Cherry Hinton, Cambridge, UK	18.09	96.31	92.35	3I-1	3
BEL024	1035–1203 cal CE	Byhau, Magileu, Belarus	43.86	97.71	97.51	3Q (New*)	4
CHRY023	1034–1162 cal CE	Cherry Hinton, Cambridge, UK	7.01	96.53	89.75	3I-1	3
EDI006	575–650 cal CE	Edix Hill, Cambridgeshire, UK	23.71	97.64	97.43	3I-1	3

**Fig. 1:**
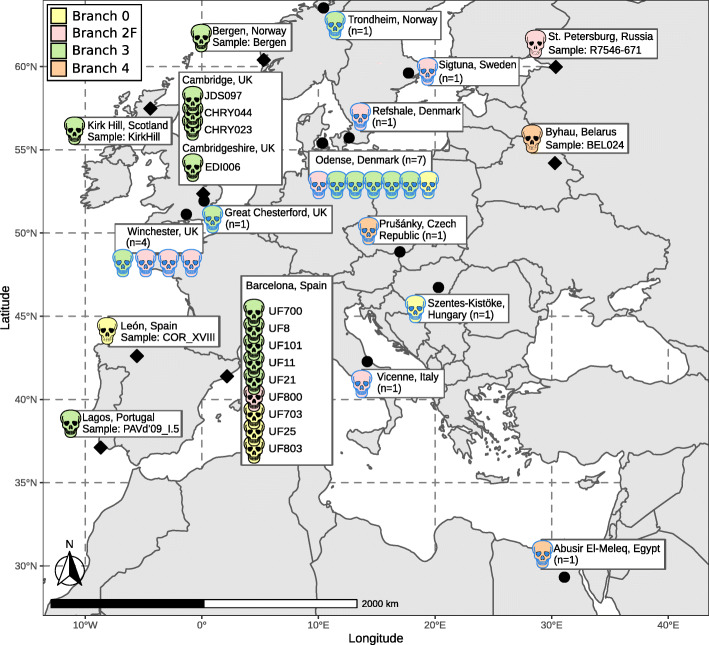
Geographic location of previously published [44, 45, 49, 96, 97] and newly reconstructed ancient genomes. The rhombuses indicate the location of the sites covered by this study; circles and skulls with blue outlines show the sites of already published *M. leprae* strains. The color of the skulls corresponds to the branches in the *M. leprae* phylogeny

We were able to detect ancient *M. leprae* DNA in libraries from 20 (~ 48.8%) individuals from ten archeological sites, spanning the period of the sixth–seventh centuries CE to the twentieth century (Additional file 1: Supplementary Note 1, Table S1 and S2) [1, 21, 30, 31, 35, 50, 52–88]; 19 of these yielded sufficient *M. leprae* DNA for genome reconstruction. The individuals positive for *M. leprae* represent a variety of geographical regions located in six modern day European countries (Fig. 1) including two leprosaria: Lagos (southern Portugal) and Barcelona (northeast Spain). From these two leprosaria, we were able to reconstruct one and nine genomes, respectively (Fig. 1). Direct dating of 14 individuals (Table 1) confirmed the archeological age estimations, except for sample PAVd’09_I.5 (Portugal) (Additional file 1: Supplementary Note 1) [1, 21, 30, 31, 35, 50, 52–88].

### Genome reconstruction

**Fig. 2:**
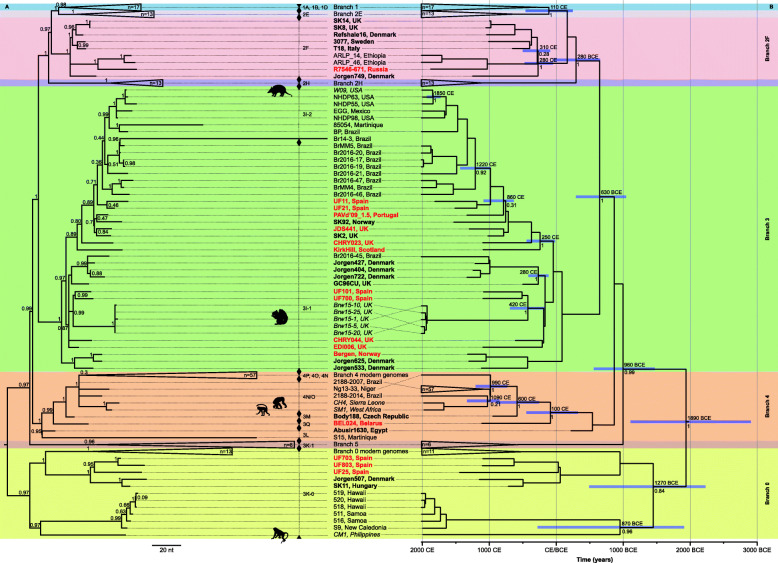
Phylogenetic trees. **A** Maximum Parsimony tree of all published modern and ancient [44, 45, 47, 49, 50, 96, 97, 108, 116–121], as well as newly sequenced leprosy strains. All ancient strains are bold, and all new ancient strains are bold and red. The bootstrap values are given as node labels (500 bs). Animal symbols and italicized labels indicate strains isolated from red squirrels (Brw15 strains), armadillos (W09), and non-human primates (CH4, SM1, and CM1). The main branches are color-coded with background boxes. **B** Bayesian Maximum Clade Credibility time-aware tree for the leprosy genomes including only genomes with at least 3-fold coverage and at least 60% of the genomic sites. Noteworthy nodes are labeled with the median estimated age (year CE/BCE) and 95% Highest Posterior Density for the age estimate (violet bars) as well as posterior probability estimate

**Fig. 3:**
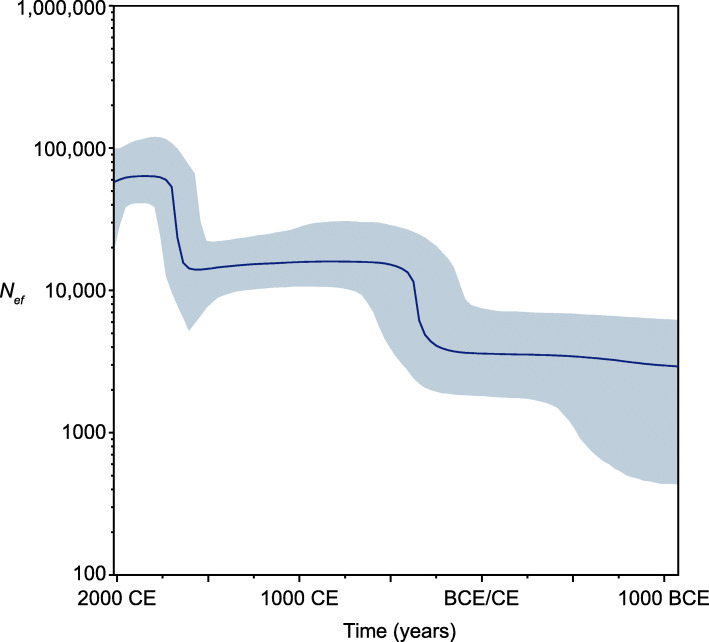
Bayesian skyline plot representing the effective population size of *M. leprae* over the period from ca. 1000 BCE to 2000 CE. Mean estimates are shown as solid line and 95% HPD limits as gray area

For 19 individuals, we were able to reconstruct *M. leprae* genomes with a 1-fold coverage for at least 60% of the genome sequence and a mean coverage of 1.46–110.61**×** (Fig. 1, Table 1). To confirm the ancient nature of DNA, we examined it for characteristic damage patterns. These are an increased frequency of cytosine to thymine base exchanges at the fragment end, which result from the deamination of cytosine to uracil during the DNA degradation process [98]. In addition, ancient DNA is highly fragmented, resulting in a short fragment length [98]. The DNA fragments used for genome reconstruction have a mean fragment length of 51–86 bp (Additional file 1: Table S2) [99–106]. The frequency of C to T base misincorporation of all non-UDG libraries results in 2–20% (Additional file 1: Supplementary Note 3, Fig. S2) [106, 107]. These genomes were sufficiently covered for a reconstruction of a Maximum Parsimony and Maximum Likelihood tree and for SNP typing, and SNP annotation (Table 1, Fig. 2A, Additional file 1: Supplementary Note 3, Fig. S3A, S3B, S4A, S4B, Table S3, Additional file 2: Table S4), [42, 45–47, 108–111]. Sixteen of these 19 reconstructed genomes have a minimum of 3-fold coverage for at least 60% of the genome sites. These 16 high-coverage genomes were used further for molecular dating by BEAST (Fig. 2B, Fig. 3, Additional file 1: Fig. S5, S6, S7) [111–115].

### Phylogenetic analysis

We combined the 19 new genomes with 177 published modern and ancient *M. leprae* genomes [44, 45, 47, 49, 50, 96, 97, 108, 116–121] to investigate the genetic affinities of the newly reconstructed genomes. All genomes from our study are placed in one of the previously defined eight branches of the *M. leprae* phylogeny [45]. Four Iberian genomes are placed in the most basal branch, branch 0 (Table 1, Fig. 2A, B, Additional file 1: Fig. S3A and S3B) [109–111]. This includes UF25, UF703, and UF803 from the leprosarium in Barcelona (Spain) and COR_XVIII (Cordiñanes, León, northwest Spain). They cluster with two medieval *M. leprae* genomes from Hungary (SK11) and Denmark (Jorgen507) presented in a previous study [45]. None of the newly reconstructed genomes is located in branch 5, but the medieval Belarusian genome BEL024 is placed in branch 4. This genome, as well as the ancient genomes Abusir1630 (Egypt) [49] and Body-188 (Czech Republic) [45], are diverged basally to most of the modern branch 4 genomes (Table 1, Fig. 2A, B, Additional file 1: Supplementary Note 3, Fig. S3A and S3B) [109–111], except for S15.

In total, 12 of our 19 (63.2%) genomes position on branch 3 (Table 1, Fig. 2A, B, Additional file 1: Fig. S3A and S3B) [109–111]: the non-leprosarium genomes from Bergen (Norway), CHRY023, CHRY044, EDI066, and JDS097 (all eastern England), and Kirk Hill (Scotland); leprosarium samples UF700 and UF101 (Barcelona, northeast Spain): and three additional leprosarium samples from the same site (UF8, UF11, and UF21), and PAVd’09_I.5 (southern Portugal).

The Bergen sample forms a sister branch to the previously published Jorgen625 (Denmark) strain [44]. Two *M. leprae* strains from Barcelona, UF101 and UF700, are closely related (SNP distance *d* = 23) and branch off basally to strains isolated from modern British red squirrels (Table 1, Fig. 2A, B, Additional file 1: Supplementary Note 3, Fig. S3A, S3B, S4A and S4B) [50, 109–111]. The samples from Kirk Hill (Scotland), and CHRY023 and JDS097 (both eastern England) are phylogenetically placed close to the previously published SK2 genome [44] from Winchester, southern England. Together with PAVd’09_I.5 (Portugal), and the three Barcelona leprosarium samples, UF8, UF11, and UF21 (fifteenth-eighteenth centuries), they form a separate cluster consecutively diverging from the evolutionary line leading to the American branch 3 cluster.

The samples R7546-671 (Russia) and UF800 (Barcelona, Spain) are placed in branch 2F (Table 1, Fig. 2A, Additional file 1: Fig. S3A and S3B) [109–111]. The Russian genome R7546-671 takes a basal position to the two modern Ethiopian genomes placed in branch 2F. The genome UF800 is located basal to the previously published ancient genomes SK8 and SK14 (Winchester, southern England) [44, 96]. The branches 2H, 2E, and 1 are so far defined by modern *M. leprae* genomes exclusively (Fig. 2A, B, Additional file 1: Fig. S3A and S3B) [109–111].

To summarize, 12 of our ancient *M. leprae* genomes are placed in branch 3, four in branch 0, two in branch 2F, and one in branch 4. The two genomes located in branch 2F are an ancient one from the leprosarium in Barcelona, clustering with medieval European *M. leprae* sequences, and the historic sample from Russia, falling basal to modern Ethiopian *M. leprae* genomes. Most of the newly reconstructed medieval *M. leprae* genomes are placed in branch 3 (Table 1) and cluster with medieval European genomes from previous studies [44, 45] all located basal to modern *M. leprae* genome clades (Fig. 2A, B). Most intriguing, the phylogenetic tree uncovered a high genetic relatedness of two medieval genomes from Barcelona (UF101 and UF 700) and the modern *M. leprae* genomes isolated from red squirrels [50] (Fig. 2A, B). One of the genomes reconstructed (from the individual BEL024) falls in branch 4, basal to most of the other branch 4 genomes (Fig. 2A, B). Lastly, four of our Iberian *M. leprae* genomes are placed in branch 0 clustering with two previously published medieval genomes [45]. This cluster is basal to modern human leprosy sequences and forms a sister clade to the genomes reconstructed from modern non-human primates.

### Genotyping and SNP effect analysis of the new strains

We also performed a more detailed analysis of the SNPs identified in our genomes including genotyping and SNP effect analysis to increase the resolution of our analysis. For the genotyping of all 19 newly reconstructed genomes, we used the method developed by Monot and colleagues [42] (Fig. 2A, Additional file 1: Table S3) [42, 45–47, 108] to allow comparability with previously published data. Briefly, there are 84 informative markers (78 SNPs and six indels in homopolymeric tracts) used for classification into the 16 SNP subtypes of *M. leprae* [42]: 1A-D, 2E-H, 3I-M, and 4 N-P. For a more straightforward application, the SNP types (SNP type 1–4) and the SNP subtypes (A-P) can be determined using a combination of three and 16 loci, respectively [42]. Deeper resolution in SNP subtyping was recently published in the SNP subtypes 3I (3I-1, 3I-2) and 3K, and the corresponding specific markers were also applied in our analysis [46]. All newly sequenced ancient genomes from branch 2F, 3, and 0 belong to the SNP subtype 2F, 3I-1, and 3 K-0 respectively (Fig. 2A). The genome (BEL024) from medieval Belarus was identified as genotype 3L according to the Monot classification [41, 42], but, phylogenetically it forms a lineage separate from the canonical 3L strains and diverge basally to the SNP subtype 3M (strain Body-188 from the Czech Republic). We propose to label this lineage as 3Q (Fig. 2A).

Using SnpEff [122], a range of 49 to 167 SNPs with potential effects were identified in the newly reconstructed genomes presented here (Additional file 2: Table S4) [44, 45, 47, 49, 50, 96, 97, 117–121, 123, 124]. Additionally, the samples have between 28 and 72 non-synonymous SNPs in coding regions. Eighteen samples have unique SNPs located within coding genes: BEL024 (*n* = 4), Bergen (*n* = 2), CHRY023 (*n* = 3), CHRY044 (*n* = 6), EDI006 (*n* = 1), JDS097 (*n* = 2), Kirk Hill (*n* = 5), PAVd’09_I.5 (*n* = 2), COR_XVIII (*n* = 5), R7546-671 (*n* = 4), UF101 (*n* = 8), UF21 (*n* = 1), UF25 (*n* = 6), UF700 (*n* = 5), UF703 (*n* = 2), UF800 (*n* = 7), UF803 (*n* = 1), and UF8 (*n* = 3); details of these unique SNPs can be found in Additional file 2: Table S4 [44, 45, 47, 49, 50, 96, 97, 117–121, 123, 124]. Six SNPs are located within coding genes that are related to virulence factors, affecting amino acid and purine metabolism (leucine synthesis), mammalian cell entry (*mce*) operons, secretion system, and cell surface components (Additional file 3: Table S5, Additional file 1: Table S6) [44, 45, 47, 49, 50, 96, 97, 108, 116, 118–124]. In parallel, only a few SNPs were found in modern strains compared to ancient strains from branches 3 and 4, for which ancient strains are basal in the branch (exception of Br2016-45 and the red squirrel strains for branch 3). Only one SNP (t954663c, *ml0805*, pseudogene) was acquired by all modern strains (Fig. 2A, from Br2016-46 to NHDP-55). A total of nine SNPs were acquired by the modern strains from branch 4 but only one led to a non-synonymous mutation in a gene coding for a phospho-N-acetylmuramoyl-pentapeptidetransferase (a1079902g, *murX*). Indel analysis was not performed due to limited genome quality.

Finally, we analyzed the specific SNPs to branch 3—the branch in which most of our genomes fall—in comparison to the other branches. We found 16 SNPs including 50% of missense mutations (Additional file 2: Table S4) [44, 45, 47, 49, 50, 96, 97, 117–121, 123, 124]. Within branch 3 we also detected a close genetic relationship between modern *M. leprae* strains isolated from red squirrels [50] and medieval *M. leprae* genomes reconstructed from two medieval Barcelona individuals (UF101 and UF700). Therefore, we investigated further into details of SNP differences and similarities of these *M. leprae* genomes. The modern *M. leprae* genomes from red squirrels differ from each other by zero to three nucleotides (0–0.11%). The two medieval *M. leprae* genomes from the individuals UF101 and UF700 from Barcelona differ in 23 nucleotides (0.82%). When comparing either the *M. leprae* genome from UF101 or UF700 to the *M. leprae* genomes from red squirrels, they are different at 41–45 positions (1.47 to 1.61%). Furthermore, the two medieval *M. leprae* genomes isolated human individuals and the five modern *M. leprae* strains from red squirrels share three specific SNPs: a synonymous SNP at position 1348383, and two non-synonymous SNPs at the positions 2271752 and 2495453. The T-to-G SNP at position 2271752 is coding for “rpoC” gene and calling glycine instead of valine, the G-to-T SNP at position is coding for the gene “mntH” and here the amino acid serine is called instead of alanine.

### Estimation of divergence times (BEAST analysis)

Bayesian time-aware phylogenetic reconstruction ( Additional file 1: Supplementary Note 3) [111–115] was performed using a relaxed molecular clock model, because the strict clock was rejected based on the coefficient of variation distribution among the branch rates [125]. The inferred phylogeny (> 0.98 posterior support for all but a few nodes; Fig. 2B, Additional file 1: Fig. S5) [111–115] supports the topology of the Maximum Parsimony tree (Fig. 2A. Additional file 1: Fig. S3A, S4A) [109–111]. The most recent common ancestor of all the sequences included in the analysis was estimated to ca. 1900 BCE (2910–1110 BCE 95% HPD, see Fig. 2B, Additional file 1: Supplementary Note 3, Fig. S5) [111–115].

Bayesian skyline estimation of demographic changes through time shows two sudden increases in *M. leprae*’s effective population size (*N*_*e*_) starting around 250 CE (± 250 years) and 1600 CE (± ca. 100 years), which potentially tie in with major changes in human connectivity (Fig. 3). In the first instance, this could include Roman expansion, and the second coincides with the arrival of Europeans in the Americas.

## Discussion

In this study, we reconstructed 19 *M. leprae* genomes from ancient individuals from across Europe (Table 1), doubling the number of available ancient genomes and providing insights into the distribution of *M. leprae* lineages in understudied regions. Using 11 of these genomes, we can evaluate *M. leprae*’s diversity within the Iberian Peninsula. The remaining eight genomes can further inform our understanding of the distribution of the pathogen within Eastern Europe, the British Isles, and Scandinavia. We now have an improved understanding of *M. leprae*’s diversity across medieval Europe; we show that the long-term predominance of branch 3 genomes seen in North-West Europe applies also to South-West Europe and that there are strains from three major branches (0, 2F, and 3) that circulate within single locations, in this case medieval leprosaria (Fig. 2A, Additional file 1: Fig. S3A, S3B, S4A and S4B). We also discovered another branch 4 strain, defined as SNP subtype 3Q, in Eastern Europe, recently most frequent in Africa [47], allowing for a better resolution of this branch’s prevalence in that region (Fig. 2A, B, Additional file 1: Fig. S3A S3B, S4A and S4B).

We were interested in understanding whether other leprosarium sites showed similar levels of strain diversity as St Jørgen (Odense, Denmark), where three different strains were found in individuals from a similar time frame [45]. Therefore, we attempted to add data from two other leprosaria and were successful in obtaining multiple genomes from individuals buried at one of them, a leprosarium in Barcelona. As demonstrated by the inferred phylogeny (maximum parsimony and maximum likelihood; Fig. 2A, B, Additional file 1: Fig. S3A, S3B) [109–111], the genomes from the Barcelona leprosarium are placed in three branches: branch 3, branch 2F, and branch 0, similarly to the genomes reconstructed from the St Jørgen cemetery. Based on radiocarbon dating, at least three Barcelona strains date to the same time period (eleventh–thirteenth centuries) and thus show that the high strain diversity identified at St Jørgen in Denmark [45] is not unique. Strains from two different branches (2 and 3) were also identified at the Winchester leprosarium [44]. This suggests that by the thirteenth century, the presence of multiple, phylogenetically distant strains in leprosaria was a common phenomenon.

As not all individuals with Hansen’s disease were buried in leprosarium contexts, and to assess circulating strains in one location over a long period of time, we also investigated the region of Cambridge. Here, we have the highest number of genomes from one geographical area from non-leprosarium contexts; two early Anglo-Saxon individuals that date prior to the widespread foundation of leprosaria in England (from the fifth–seventh centuries: Great Chesterford; Edix Hill, EDI006), and two from the tenth–twelfth century (Cherry Hinton, CHRY023, CHRY044) and one of thirteenth century date (Hospital of St John, JDS097) when leprosaria are known to exist in this region of England [1]. Interestingly, all belong to branch 3 and thus show low diversity (Fig. 2A, Additional file 1: Fig. S3A, S3B, S4A and S4B), even though branch 2 strains were present in England by at least the thirteenth century [44] and possibly as early as the eleventh century, based on SNP typing [126].

Although it is necessary to study a greater number of individuals from non-leprosarium contexts from the same location, the high strain diversity found in the leprosarium at Barcelona raises interesting questions about the nature of leprosarium sites and whether individuals entering some of these institutions originated from diverse locations. It is thought that most leprosaria were founded to serve local people but some possibly also admitted the “wandering leprous,” meaning individuals with no accommodation alternatives [23, 127], which could have included pilgrims [128]. Furthermore, high-status leprosaria may have attracted individuals from more diverse backgrounds, who perhaps had more opportunities to travel during their lifetime (e.g., pilgrimage, military encounters, trade), before and while they resided in those specialized hospitals. The surviving documentation from the leprosarium at Barcelona is particularly revealing; although initially founded for people with “leprosy” in towns, the account books from the end of the fourteenth century show that many non-local people who had travelled or undertook pilgrimage, were present there [67]. In addition, there are also a number of accounts and tales of people undertaking a pilgrimage specifically in the hope of obtaining a cure for “leprosy” [2].

Questions surrounding the importance of pilgrimage in the spread of the disease have already been raised through the identification of the “Pilgrim Burial” at Winchester and the mobility of people with the disease might have been underestimated in the past [128]. Barcelona was likely important for those travelling to Santiago de Compostela, a major pilgrimage destination in medieval Christian Europe [128]. Further work in this area might reveal the important role of different types of mobility in the spread of the disease, as has been attempted for other past infections [129, 130]. To investigate the potential role of leprosaria as “diversity pools,” the questions of whether there is low diversity in surrounding populations from Odense and Barcelona and if there is high diversity in leprosarium sites in Cambridge’s region of East Anglia in eastern England need to be assessed. Assessing evidence in Norwich, an important East Anglian port town around 100 km northeast of Cambridge, where there is evidence for Hansen’s disease in skeletons from a cemetery possibly associated with a leprosarium [131] might be revealing [132]. There are also other (non-leprosarium) sites in East Anglia that have revealed skeletons with Hansen’s disease [1]. It may also be beneficial to assess individuals from a newly excavated leprosarium, St Leonard’s in Peterborough, approximately 65 km northwest of Cambridge. The use of stable isotope analysis to explore the origin and mobility histories of those buried there at a population level in a leprosarium context would also be beneficial. Further work on historical sources from leprosaria and other documentation might also be able to reveal more about the distances and routes that people travelled.

While we identified strains from multiple branches, branch 3 genomes were most common, a trend also seen in our previous work [45]. Although future research needs to assess more individuals with Hansen’s disease from regions where we have limited or no genomes, it is becoming increasingly evident that branch 3 strains were both widespread and predominant in medieval Europe, especially in Western regions. For England and East Anglia in particular, we can confirm that this branch was likely dominant throughout the disease’s known 900-year history (approximately 500 CE–1400 CE) as suggested by Inskip and colleagues [133]. If we consider the presence of branch 3 genomes at the leprosarium in Barcelona, we can also observe a long history of branch 3 strains from around 1100 CE to the eighteenth century. Combined, the Cambridgeshire and Barcelona data show that branch 3 strains have been circulating in Western Europe for over 1200 years, with little genetic variation; they differ in only around 1100 positions of the circa 3.2 Mbp *M. leprae* genome (Additional file 2: Table S4) [44, 45, 47, 49, 50, 96, 97, 117–121, 123, 124]. Modern branch 3 strains closely related to them can still be found in the USA and South America [44, 45, 47] as well as in two animal reservoirs, further reflecting the complex history of this branch as well as the previously observed slow-evolving nature of the pathogen [44, 45, 134]. Overall, the 17 medieval and two early modern European genomes of branch 3, including 12 of our new strains, provide a so far unique resolution for the history and past dissemination of this branch. This research has refined our information about the relevance of link to population dynamics and the spread of *M. leprae* (Fig. 2A, B, Additional file 1: Fig. S3A, S3B and S4A, S4B).

For Eastern Europe, the identification of a novel SNP subtype, here named 3Q, in an individual from Belarus (9th to 11th CE) which sits in branch 4 basal to the Body188 genome from the Czech Republic [45], might reflect a long-term presence of strains from this branch in the region. Currently, branch 4 is composed of six SNP subtypes: 3L [49], 3M [44, 45], 4N/O [135], 4N, 4O, and 4P [47]. While the three SNP subtypes 4N, 4O, and 4P are well described in modern samples, they have yet to be identified in ancient samples. Conversely, SNP subtypes 3L and 3M strains are very rare in modern *M. leprae* samples, being mainly identified on islands (such as Martinique or New Caledonia [42]) and mostly described from ancient remains from Eastern Europe and North Africa [42–45, 49]. In comparison to strains of other branches circulating in medieval Europe, the SNP subtypes 3M and 3L seem to be less successful in surviving in the modern world. This discovery of a new SNP subtype in ancient remains points to a potential loss of diversity over time. For example, it is possible that modern hygienic practices or cross-immunity/competition with other diseases such as tuberculosis have had an impact on diversity [1, 26, 38]. This is also reflected in the poor resolution of the basal structures of branch 4 in the phylogenetic tree. However, the apparent loss of diversity might also be an artifact of our limited knowledge of modern diversity. Overall, it highlights the importance of future investigations in medieval Eastern Europe and North Africa in order to trace back the evolutionary history of branch 4 and capture its past and present diversity.

Interestingly, we observe a close phylogenetic relationship between the nineteenth and twentieth century strain from Russia to those in modern Ethiopia [47] (Fig. 2A, B, Additional file 1: Fig. S3A, 3B, S4A and S4B). A potential explanation for this relationship may come from a series of historic events, pointing to direct contacts between Russia and Ethiopia at the end of the nineteenth century and the beginning of the twentieth century: In the late nineteenth century, Russian settlers arrived in Ethiopia in order to establish “New Moscow” in the region of modern Djibouti [136, 137]. Although the Russian settlers were forced to leave by the French army in 1889, Russian-Ethiopian relations continued and resulted in Russian support of the Ethiopians in the Ethiopian-Italian war [137]. After the victory of Adwa in 1896, which secured the independence of Ethiopia, formal political relations between Russia and Ethiopia started. These historic interactions may have caused the exchange of pathogens between the regions, including the *M. leprae* strain presented here. Historic reports confirm the prevalence of Hansen’s disease in Russia in the late nineteenth to early twentieth century [138, 139] (Additional file 1: Supplementary Note 1) [1, 21, 30, 31, 35, 50, 52–88], further supporting potential transmissions.

In concordance with previous studies focusing on Northwest Europe [44, 45, 96], our results confirm that a high *M. leprae* diversity is also present in other parts of medieval Europe. Despite our new information on strain diversity and increased resolution regarding the reconstruction of the history of Hansen’s disease, we still cannot resolve its origin. The lack of ancient samples from potential source areas and older time periods prevents us from favoring one of the two models proposed by Schuenemann and colleagues [45]. Even the genome Abusir1630 from Ancient Egypt [49], so far the only ancient genome from outside of Europe, is located basal to branch 4 and therefore cannot contribute to discussions of the origin of the other branches present in medieval Europe. In comparison, regions with a significant prevalence of Hansen’s disease today have different but consistently lower levels of strain diversity than discovered for medieval European leprosaria as well as the strain diversity estimated for the entire medieval European continent. However, we observe different levels of modern strain diversity in distinct geographic regions: higher levels are present in some endemic countries such as Brazil (branches 1, 3, and 4) and India (branches 1 and 2) while lower levels exist on small islands such as Madagascar (only branch 1), as well as in Ethiopia (branch 2), or West African countries (branch 4) [44, 45, 47, 50, 119]. This modern diversity variation may be linked to past migrations resulting in new introductions of strains, potentially European ones.

While it is always difficult to relate specific events to increased diversity due to the wide time ranges associated with archeological dating and their use in molecular dating approaches, we see two significant episodes of *M. leprae* population expansion in the Bayesian skyline plot (Fig. 3) that coincide with important shifts in human connectivity. The first date range 250 CE ± 250 years broadly covers that of Roman conquest and expansion, while the later date range, 1600 CE ± 150 years, is consistent with rapid advances in knowledge and technology in the late Medieval period which culminated with the arrival of Europeans in the Americas and the beginning of regular long distance (transatlantic) trade. Historical and archeological data have already highlighted a link between the increasing expansion of the Roman Empire and the spread of Hansen’s disease [1, 43, 140] and our results support this hypothesis. Coincidently, it is from this period that we start to see some of the earliest evidence in the osteological record, especially in Europe [1]. For the Late Medieval period, genetic data show the link between European *M. leprae* branch 3 strains and those currently circulating in the Americas [44, 45, 134, 141]. Modern armadillos harbor the same branch 3 strains as those identified in ancient European samples, showing a direct link between the two regions [108]. In both situations, increased global connectivity may have introduced new strains to regions that already had an endemic strain, but the introduced strains outcompeted them or found new hosts. This is a situation common today with rapid and frequent global travel being a key part of the global economy [142].

While individuals in populations that have never experienced a particular infectious disease may be more vulnerable to new incoming diseases [143], radical changes and disruptions in a social organization often associated with colonization events, like those explored here, are also key in increasing indigenous populations’ vulnerability. Important factors can include malnutrition, conflict, breakdown of social networks, forced labor/slavery, or similar conditions of stress [144, 145]. As Hansen’s disease outcomes are strongly dependent on the immune response of potential hosts [146], its arrival into immunologically compromised populations, perhaps in poor communities, may be significant here and these factors may have provided greater opportunities for the bacterium to spread and multiply, explaining the increase in effective population size.

Bayesian molecular clock inference provided us with estimates of the age of the most recent common ancestors and divergence of branches of *M. leprae* [114]. Our evolutionary timescale estimates are concordant with some previously published [45, 47], and a discrepancy with the latest published estimate [49] calls for cautious interpretations and further investigation in the future. However, the 95% credibility intervals for the age of the most recent common ancestor estimates do overlap (so they do not differ significantly). Nonetheless, with the higher resolution time-aware phylogeny, we can now refine the potential estimates for the time of the interspecies transmission between red squirrels and humans, and contribute to “One Health” that explores links between humans, other animals, and the environment. In the future, this multidisciplinary and multimethod-driven approach will be key to investigating leprosy’s evolutionary history to understand past and recent spread and transmission of Hansen’s disease. This important approach combines evidence from archeology and modern genetics and can help us understand the importance and relevance of red squirrels in spreading the pathogen in the past, and what this means for the present. Work by Avanzi and colleagues [50] and others [45, 47] showed that the closest sequenced genome to that in modern squirrels was retrieved from a fifth to sixth century male individual (SK2) from Great Chesterford, Essex (eastern England and close to London). However, with our data we identified additional ancient genomes from Iberia and England with a close relationship to *M. leprae* strains isolated from modern red squirrels from England in branch 3 (Fig. 2A, B, Additional file 1: Fig. S3A, S3B, S4A, S4B and S5) [109–115]. While the Edix Hill genome is placed more basal to that identified at Great Chesterford, the new genomes from Barcelona are more closely related to those recovered from red squirrels. Comparing these genomes with each other, we detected low differences in the *M. leprae* DNA sequences isolated from these individuals. Furthermore, we can find one synonymous and two non-synonymous SNPs in coding regions (Additional file 2: Table S4) [44, 45, 47, 49, 50, 96, 97, 117–121, 123, 124], that are only shared between the genomes from Barcelona and the genomes of modern red squirrels, further supporting their close relationship. With this finer resolution, we can now estimate the split time of all human- and squirrel-associated genomes on branch 3 to the fifth century CE (95% HPD 160 CE–690 CE; Fig. 2B). Hence, we suggest that this specific leprosy strain was potentially transmitted slightly earlier than previously thought [133]. This had already happened in Late Antiquity/the Early Medieval period (200 CE–700 CE). However, our data do not support a clear indication of the nature of the transmission, whether that was anthropologically or zoonotically. While significant human-squirrel interactions are reported in the High and Late Medieval Periods (1100 CE–1400 CE) [45, 147], little is known about such interactions in the preceding periods, although squirrel pelts were certainly produced for the Viking Age markets [148]. Given limited knowledge on the squirrel fur trade during the Viking period, including how animals were obtained and processed, this is an avenue that should be explored.

## Conclusion

In conclusion, our new ancient genomes from Europe, including an eastern European one with a new SNP type from Belarus, support previous observations of high diversity of *M. leprae* in the past by finding a similar phylogeography in Europe. In addition, we observe a high diversity at a leprosarium, indicating that leprosaria received infected individuals with diverse strains and from various geographic backgrounds. New estimates on the past population diversity of this pathogen further allow insights into its global history in relation to major historic events. Although we were able to refine our understanding of the interspecies transmission of *M. leprae* between red squirrels and humans, this important One Health aspect cannot be resolved by studying only ancient human strains. With the inclusion of ancient animal samples, the picture of *M. leprae*’s evolutionary history enables new avenues of research for approaching this aspect in future studies.

## Methods

### Material: sample information

In our study, we analyzed 81 samples from 41 individuals from the early medieval to the early modern period (Additional file 1: Table S1) [1, 21, 30, 31, 35, 50, 52–88], to address questions about intra- and interregional genetic diversity of ancient *M. leprae*. Predominantly, we included samples from regions where no genome-wide data were as yet available. As part of early screening work, we also assessed one Scottish Bronze Age sample (Additional file 1: Supplementary Note 1) [1, 21, 30, 31, 35, 50, 52–88]. Unfortunately, it had a very low DNA preservation, but because of the destructive nature of the process, and the temporal importance of the sample, ethically speaking it is important to mention this negative result. It is therefore fully excluded from all work presented here. However, it should be noted that *M. tuberculosis* complex DNA was identified by GM Taylor, but could not be replicated [15]. All other individuals studied here, except one (JDS097), were previously associated with Hansen’s disease due to their archeological context or due to pathological lesions compatible with a diagnosis of Hansen’s disease (Additional file 1: Supplementary Note 1) [1, 21, 30, 31, 35, 50, 52–88]. We investigated bone and/or tooth samples of these 41 individuals to confirm their archeological association with leprosy at the genetic level through the reconstruction of ancient *M. leprae* genomes.

### Methods: sample processing

#### DNA extraction

DNA extraction and pre-amplification steps of all leprosy samples analyzed in this study were undertaken either in the cleanroom facilities at the University of Tübingen, the University of Zurich, the University of Cambridge, or the University of Tartu. Post-amplification steps were performed in separate DNA laboratories at the University of Tübingen, the University of Zurich, University of Cambridge, the Max Planck Institute for the Science of Human History (MPI-SHH) in Jena, and the University of Tartu. All laboratories fulfil the requirements for ancient DNA research [149, 150]. To minimize the risk of potential contamination, all samples were specially pre-treated before DNA extraction (Additional file 1: Supplementary Note 3) [151]. For DNA extractions, we applied a well-established guanidine-silica-based extraction protocol developed for ancient DNA work [151] and used 30–120 mg of bone powder. For the DNA-extraction step, positive and negative controls were produced; positive controls to determine whether the DNA was successful or not, negative controls to identify potential contamination. The negative controls were carried along with all laboratory experiments and were also sequenced, the positive control till the first step of library preparation.

#### Library preparation

In this study, we produced double-stranded non-UDG and UDG-treated, as well as single-stranded DNA libraries (see Additional file 1: Supplementary Note 3) [98, 152–155]. The double-stranded DNA libraries produced in Tübingen and Zürich were used for a screening capture, an in-solution capture approach, used to enrich the DNA for three specific leprosy genes and direct sequencing (Additional file 1: Supplementary Note 3) [156, 157]. The samples processed in Tübingen were enriched additionally for human mitochondrial DNA [44, 156, 157] (Additional file 1: Supplementary Note 3) [156, 157]. Genome-wide enrichment was either performed by an array capture approach [158] and applied to all Tübingen samples, except the leprosy samples from Barcelona; or by an in-solution genome-wide enrichment using specifically designed RNA baits [119, 159], which was applied to the Barcelona samples as well as the samples processed in Cambridge, Jena, and Tartu (Additional file 1: Supplementary Note 3) [159].

#### DNA sequencing

DNA was sequenced either at the MPI-SHH Jena, the Functional Genomics Center at the University of Zurich (FGCZ), or the Institute of Genomics Core Facility at the University of Tartu (UTIG).

#### Downstream analysis of sequencing data

For a detailed description of the downstream analysis, see Additional file 1: Supplementary Note 3 [44, 49, 96, 97, 99–108, 116–118, 120, 121, 160]. Briefly, the sequenced DNA was screened for positive *M. leprae* reads using the EAGER pipeline [99]. For those samples containing sufficient authentic DNA reads mapping against the *M. leprae* genome, the complete ancient genome was reconstructed using software integrated into the EAGER pipeline [99] (Additional file 1: Supplementary Note 3) [100–107]. Sequencing reads of previously published samples included in our study were processed identically (Supplementary Note 3) [44, 45, 47, 49, 50, 96, 97, 100–108, 116–121, 160]. In addition mitochondrial haplogroups were determined for those libraries included in mitochondrial DNA capture and the data of the directly sequenced libraries were used for molecular sex determination (Additional file 1: Supplementary Note 3, Table S1) [99, 161–166].

#### SNP typing, SNP alignment, and SNP effective analysis

We performed SNP typing [41, 42], SNP alignment [123], and SNP effect analysis [122] for those samples in which we were able to reconstruct an ancient genome with a 1-fold coverage of at least 60% of the genome. These genomes were also included in the reconstruction of a Maximum Parsimony tree (Fig. 2A, Additional file 1: Fig. S3A, S4A and Supplementary Note 3) [109–111] and Maximum Likelihood tree (Additional file 1: Fig. S3B and S4B) [109–111]. For SNP typing, the genomes were first aligned to the reference genome Mycobacterium leprae TN (NC_002677.1). In a second step, branch-specific SNPs were detected by comparing all genomes, and third step, specific SNPs for ancient genomes were determined by comparing the modern and ancient genomes of particular branches.

#### Estimation of divergence time (BEAST analysis)

Only the high-quality samples with a minimum 3-fold coverage of at least 60% of the genomic sites were used for Bayesian time-aware phylogeny and past population dynamics inference with BEAST [114] (Additional file 1: Supplementary Note 3, Fig. S5, S6 and S7) [111–115]. The resulting Skyline plot shows changes in the effective population size (*N*_*e*_) through time (Fig. 3). Although *N*_*e*_ is not an estimate of the actual census size of the population, it should well reflect the relative changes in the number of transmitted bacteria.

## Supplementary Information

**Additional file 1:** Supplementary Information **Supplementary Note 1** Archaeological information for the sites and samples. Radiocarbon dates. **Supplementary Note 2** Radiocarbon Dating **Supplemetary Note 3** Applied Methods. **Fig. S1.** Sample Processing and Genome-wide analyses **Fig. S2.** Combined damage profiles. **Fig. S3.** Phylogenetic trees of 1-fold covered genomes. **Fig. S4.** Phylogenetic trees of 3-fold covered genomes. **Fig. S5.** Bayesian Maximum Clade Credibility time-aware tree for the leprosy genomes. **Fig. S6.** Date Randomization Test for the M. leprae dataset. **Fig. S7.** TempEst analysis for the M. leprae dataset. **Table S1.** Sample information. **Table S2.** Eager Report. **Table S3.** SNP subtyping. **Table S6.** Unique SNPs and virulence factors.

**Additional file 2: Table S4.** Result table of the SNP effect analysis.

**Additional file 3: Table S5.** SNP distance matrix.

## Data Availability

Raw sequencing data are available on NCBI (BioProject ID: PRJNA721828).
