# Supplementary material for: Mycobacterium leprae diversity and population dynamics in medieval Europe from novel ancient genomes
Source: BMC Biol. 2021 Oct 5;19:220. doi: 10.1186/s12915-021-01120-2 (PMC8493730; doi:10.1186/s12915-021-01120-2)
Supplement: Supplementary file 1 — Additional file 1: Supplementary Information Supplementary Note 1 Archaeological information for the sites and samples. Radiocarbon dates. Supplementary Note 2 Radiocarbon Dating Supplemetary Note 3 Applied Methods. Fig. S1. Sample Processing and Genome-wide analyses Fig. S2. Combined damage profiles. Fig. S3. Phylogenetic trees of 1-fold covered genomes. Fig. S4. Phylogenetic trees of 3-fold covered genomes. Fig. S5. Bayesian Maximum Clade Credibility time-aware tree for the leprosy genomes. Fig. S6. Date Randomization Test for the M. leprae dataset. Fig. S7. TempEst analysis for the M. leprae dataset. Table S1. Sample information. Table S2. Eager Report. Table S3. SNP subtyping. Table S6. Unique SNPs and virulence factors. [file 12915_2021_1120_MOESM1_ESM.pdf]

# Supplementary Information

## *Mycobacterium leprae* diversity and population dynamics in medieval Europe from novel ancient genomes

Saskia Pfrengle, Judith Neukamm, Meriam Guellil, Marcel Keller, Martyna Molak, Charlotte Avanzi, Alena Kushniarevich, Núria Montes, Gunnar U. Neumann, Rezeda I. Tukhbatova, Nataliya Y. Berezina, Alexandra P. Buzhilova, Dmitry S. Korobov, Stian Suppersberger Hamre, Vitor M.J. Matos, Maria T. Ferreira, Laura González-Garrido, Sofia N. Wasterlain, Célia Lopes, Ana Luisa Santos, Nathalie Antunes-Ferreira, Vitória Duarte, Ana Maria Silva, Linda Melo, Natasa Sarkic, Lehti Saag, Kristiina Tambets, Ella Reiter, Philippe Busso, Stewart T. Cole, Alexei Avlasovich, Charlotte A. Roberts, Alison Sheridan, Craig Cessford, John Robb, Johannes Krause, Christiana L. Scheib, Sarah A. Inskip and Verena J. Schuenemann.

# Table of Contents


Supplementary Note 1: Archaeological information of the samples 4

1.1 Nonneseter site, Bergen, Norway 4

1.2 Saint Petersburg, Russia 5

1.3 Kirk Hill (also published as Kirkhill), St Andrews, Scotland, U.K. 6

1.4 Lagos, Portugal 7

1.5 Cordiñanes de Valdeón, León, Spain - COR_XVIII 9

1.6 Belarus - Studenka necropolis 10

1.7 Edix Hill (Barrington A), Cambridgeshire, UK. (BAEH89/90/91) 12

1.8 Church End, Cherry Hinton, Cambridgeshire (HAT358/1). 13

1.9 The Hospital of St John the Evangelist, Cambridge, Cambridgeshire (JDS10) 14

1.10 The Hospital of Sant Llàtzer or Santa Margarida, Barcelona, Spain 15

1.11 Blokhuizen, The Netherlands. 18

1.12 Santarém, Portugal 19

1.13 Beja, Portugal 20

1.14 Travanca, Portugal 21

1.15 Dryburn-Bridge, East Lothian, Scotland 21

1.16 Santa Lucia, Spain 22

1.17 Kich Malka 23

Supplementary Note 2: Sample Processing and Genome-wide analyses 25

2.1 Sampling 26

2.2 Library Preparation 27

2.2.1 Double-stranded DNA Libraries 27

*2.2.2 UDG-treated DNA Libraries* 28

*2.3 Enrichment strategies* 29

*2.3.1 Human mitochondrial capture* 29

*2.3.2 Mycobacterium leprae gene screening* 29

*2.3.3* Mycobacterium leprae *genome-wide enrichment* 29

*2.4 DNA sequencing* 30

*2.5 Genome-wide analysis - Read processing, mapping, and variant calling* 31

*2.5.1 Processing of published samples* 32

*2.5.2 SNP typing* 32

*2.5.3 SNP alignment and SNP Effect analysis* 32

*2.5.4 Phylogeny* 33

*2.5.5 Estimation of divergence time (BEAST analysis)* 34

*2.5.6 Temporal signal* 35

*2.6 Human mitochondrial DNA analyses and molecular sex determination* 35

*Supplementary Figures* 37

*Supplementary Tables* 44

*References* 56

# Supplementary Note 1: Archaeological information for the sites and samples

### 1.1 Nonneseter site, Bergen, Norway

*Stian Suppersberger Hamre*

The tooth (left first upper molar) from Bergen, Norway, comes from the Nonneseter site. The site was excavated in 1872 and 1891 [52] but the recovered skeletons were never analysed and the remains were not collected according to modern standards. All the skeletal remains were lumped together and when the osteological material was first analysed in 2006 [53], the sample consisted of several large wooden crates with commingled material. Skeletons were excavated from the graveyard on the north and the south side of the convent as well as inside the convent church. A large number of graves were found but the exact number is unknown. The estimated minimum number of individuals is 84 adults and 27 sub-adults. The Nonneseter convent was established around 1150 and secularised in 1528. Thus, it is reasonable to assume that the burials took place during this period. There are very few documented cases of leprosy from this period and this partial facial skeleton was the first described case of lepromatous leprosy from medieval Bergen. Due to too little being preserved of this individual, a reliable determination of sex is not possible and the person’s age at death cannot be estimated more accurately than adult. The signs of lepromatous leprosy, however, are there. This individual shows resorption of both the anterior and posterior walls of the alveoli for the incisors and canines and pitting which has spread all across the hard palate and perforated the posterior-sagittal portion of it. Pitting is also present on the nasal floor.

## 1.2 Saint Petersburg, Russia

*Nataliya Y. Berezina (I), Alexandra P. Buzhilova*

The sample from the individual from St. Petersburg, Russia (collection ID 7546-671) is a part of the collection of the radiologist and one of the founders of paleopathology in Russia, D.G. Rokhlin. His collection consisted of archaeological (from Bronze Age) and modern (till the mid-20$^{th}$ century) individuals with various bone manifestations of pathologies, including infectious diseases. Unfortunately, after his death in 1981, the collection was improperly stored resulting in the loss of many of the labels with sample identification information. Sample #7546-671 belongs to this collection, with the radiocarbon date of the sample shows that it is part of the modern material (19$^{th}$-20$^{th}$ century).

Sample #7546-671 is a skull of an adult female with clear bone manifestations of leprosy: rhinomaxillary remodeling, rounding of the margins of the piriform aperture, alveolar recession intravital tooth loss and cribra orbitalia which may result from insufficient nutrition and/or chronic inflammation [54]. This woman may be from a medical collection obtained from a 'leper' colony cemetery. A scientific study was undertaken to establish the situation of lepers in the Russian Empire in 1894 [55]. There was only one 'leper' colony near St-Petersburg, named Krutye ruchii. This was a colony with barracks for singles, houses for families, doctors, staff and services. There were 85 people in the colony (47 men and 38 women) in 1911, and 88 people (52 men and 36 women) were treated in the colony in 1912. The size of the colony did not increase much over time, remaining approximately at the same level. In the 1930s, many famous local doctors worked and conducted research on Hansen's Disease here. It can be assumed that the examined skull could have been brought to St. Petersburg for a scientific medical collection.

### 1.3 Kirk Hill (also published as Kirkhill), St Andrews, Scotland

*Alison Sheridan, Charlotte A. Roberts, Philippe Busso, Charlotte Avanzi and Stewart T. Cole*

The sampled individual, an adult female (SK 275A), comes from an early medieval inhumation cemetery, associated with St Mary's Church (formerly known as the Church of the Blessed Mary of the Rock) on the east Scottish coast at St Andrews. The sample, both for DNA analysis and for radiocarbon dating, was taken from the left petrous temporal bone.

A rescue excavation undertaken in 1980–81 in response to coastal erosion of the cemetery by the sea, uncovered the remains of over 300 individuals [30]. Skull SK 275A was found resting on the torso of an unrelated individual, SK 275, and it had clearly been redeposited among the earliest layers of graves, which had previously been radiocarbon-dated to as early as the 7$^{th}$ century CE (*ibid.*). The radiocarbon date of cal CE 1030–1155 (95.4%; SUERC-91431 [GU54472], 940±25 BP, $d^{13}C$‰ -20.0, $d^{15}N$‰ 11.3, C/N ratio 3.4) that was obtained for SK 275A suggests a high level of disturbance from centuries of intercutting graves.

The earliest reference to the existence of a hospital in St Andrews dates to the second quarter of the 12$^{th}$ century (*ibid.*, 310), but from 1178 there are references to a leprosy hospital at St Nicholas, to the south of the medieval city. The occurrence of other chronic diseases in the people buried within the Kirk Hill cemetery may suggest that this could have been associated with an early hospital site, but the mixed population of males, females, and children is otherwise that of a general lay burial ground. This new dating evidence indicates that even as late as the 12$^{th}$ century, people with leprosy were not yet segregated from the general population, at least in death. This has been noted in the majority of instances where skeletons with leprosy have been found, including in the UK [1].

The skull had previously attracted attention as constituting rare early evidence for leprosy in Scotland, the relevant features having been identified osteologically by Dr Dorothy Lunt [31]. There are very few skeletons identified with leprosy that have been identified in Scotland. The skeleton was probably female and at death was a young adult aged between 25 and 35 years, but this was based on dental wear patterns [56], which as a sole age estimation method is not reliable. There was inflammatory pitting of the nasal and oral surfaces of the palatal bones and the alveolar bone around the maxillary incisor tooth sockets. The anterior nasal spine was absorbed. The posterior aspect of the palatal and maxillary bones showed bone loss.

DNA was extracted from sample taken from the petrous portion of the temporal bone and screened for the presence of *M. leprae* and *M. lepromatosis* as described elsewhere in this work. Only *M. leprae* was detected and the genome sequence is reported here.

### 1.4 Lagos, Portugal

*Maria Teresa Ferreira, Sofia Wasterlain and Vitor M.J. Matos*

The buildings of the Lagos leprosarium and a modern urban dump dated from the 15$^{th}$ – 17$^{th}$ centuries were identified during the archaeological survey previous to the construction of an underground car park located in Valle de Gafaria (Lagos, Portugal) [57, 58]. The area was located outside the line of medieval walls that protected the city of Lagos. It was necessary to carry out a meticulous archaeological excavation that, in addition to exposing the leprosarium buildings, allowed the identification of a cemetery associated with it, as well as recovering the skeletal remains of 158 African enslaved individuals from the nearby dump [57–59].

At the cemetery associated with the leprosarium, eleven adult individuals were exhumed: two females, two males, and one individual of unknown sex [21]. Five of these individuals

showed several bony lesions. After the differential diagnosis (based both on macroscopic and radiological analyses) leprosy was the most probable diagnosis for two individuals (PAVd'09_I.5, adult female, and PAVd'09_I.34, adult male). As for the remaining three individuals, one was diagnosed with treponematosis, another one with brucellosis, and one with Legg-Calvé-Perthes disease [21].

Historical sources reveal that medieval leprosaria admitted not only leprosy patients but also the very poor, mentally disabled, and people suffering from other potentially stigmatizing diseases (such as syphilis, tuberculosis, among others). In fact, the leprosarium location, outside the city walls and nearby the urban dump where the corpses of slaves were discarded testifies the social exclusion of these individuals in this period and location [21, 58].

For the present study, the young adult female skeleton, with the reference PAVd'09_I.5, was analysed. This skeleton is relatively well preserved, but with both femurs, right tibia and fibula fragmented, and left tibia and fibula, and feet bones absent [21]. Several pathological signs compatible with a diagnosis of probable Hansen's Disease were observed: rhinomaxillary lesions, including rounding of the pyriform margins, complete resorption of the nasal spine, and perforated palate; woven bone deposits at the humerus, radius, ulnae and hand phalanges; porosity and osteolytic lesions at carpal and metacarpal bones; hand phalanges with erosive/destructive lesions on distal extremities [21]. That is, this female skeleton presents abnormal bone formation, abnormal bone destruction, sclerosis on lytic margins, bilateral and symmetrical lesions, and both axial and appendicular involvement [21]. Radiocarbon dating was performed on a tibial bone fragment while the maxillary bone fragment provided possible results for our ancient DNA study. The $^{14}C$ dates the sample to the 13$^{th}$ to 14$^{th}$ (Table 1, Additional file: Supplementary Note 3, Table S1) century. Hence, radiocarbon dating dates the individual 100 - 200 years further back in time than the Lagos leprosaria.

### 1.5 Cordiñanes de Valdeón, León, Spain (COR_XVIII)

Laura González-Garrido, Sofia N. Wasterlain, Célia Lopes

According to historical documentary sources, leprosy was a relatively common disease in the medieval Iberian Peninsula [60]. In the 13th century, leprosy was widespread in the north of Spain [61]. This is documented by the presence of 24 leprosy hospitals established in Asturias on the main pilgrim routes to Santiago de Compostela (Galicia) and Santo Toribio de Liébana (Cantabria).

The Barrejo medieval necropolis (12th - early 13th century) is located in the valley of Valdeón, National Park of Picos de Europa, corresponding to the locality of Cordiñanes de Valdeón (COR), in the province of León (northwestern Spain). This location is delimited and relatively isolated by the Cantabrian Mountains. From Barrejo necropolis 27 individuals have been recovered: 25 adults (18 males and seven females) and two non-adults (5-8 years old). For the present study, the adult male skeleton, with the reference COR_XVIII was analysed. This individual was inhumed in a supine position with upper limbs at the sides of the torso on a west-east axis, in a stone-lined grave and lacking grave goods. The skeleton COR_XVIII is relatively well preserved although its structure was affected by chemical diagenesis due to the necropolis proximity to the river Cares. There were also some osteological elements missing, namely the right side of the mandible, the proximal epiphyses of both fibulae, two vertebrae, feet and hand bones (two tarsals, three carpals, two metacarpals and, two hand and 21 feet phalanges).

COR_XVIII shows destructive rhino-maxillary alterations, complete resorption of the nasal spine and perforated palate; bilateral and symmetrical periostitis on tibiae and fibulae and cortical periosteal reaction, and right fibulae subperiosteal bone reaction. There are no destructive lesions on distal phalanges of the hands or feet. The radiographic analysis of the tibiae and fibulae shows the reduction in the size of the medullary cavities, particularly in tibiae, and maintenance of cortical thickness. Differential diagnosis based both on

macroscopic and radiological analyses of the lesions point to an early stage of leprosy for COR_XVIII [62].

In the medieval period, a small commercial exchange [63] and different pilgrim routes (Tolivar, 1966) could bring infirmed people to the small village of Cordiñanes de Valdeón. The burial ritual of COR_XVIII was equivalent to that of other individuals buried in this necropolis, which suggests that people suffering Hansen's Disease were not necessarily stigmatised in death.

### 1.6 Belarus - Studenka necropolis

Alena Kushniarevich

Sample BEL024 originates from the burial mound N96 of the Studenka necropolis dated 10-12$^{th}$ century CE, near Studzenka Village, Byhau region, Mahileu distr (Mahileu Dniepr river region). The site was excavated in 2015 by Alexei Avlasovich. The archaeological dating of the mound is hindered by the absence of ceramic vessel's crowns. However, the funeral rite and presence of the circular ceramics suggest that the mound was erased not earlier than the end of 10$^{th}$ or beginning of 11$^{th}$ century CE.

The skeletal material was investigated by Vladimir Shipillo. A skull (with mandible) belonged to a male individual 25-30 years old. The skull is characterized by undeveloped relief with a relatively inclined forehead; large values of longitudinal and small values of transverse head diameters; pronounced dolichocranic (cranial index=72 mm); average values of nose index (57.8 mm); average values of orbital index (77.5 mm). The skull had an ovoid shape. The face was orthogonal (face protrusion index=91.9 mm). The stature of the individual was calculated using Pearson and Li formulae applied to the right femur and estimated to 162.09 cm.

The Studenka necropolis belongs to the Ancient Rus epoch. The mound burial is located on the left bank of Greza River, the right inflow of Drut River, 1.5 km north-east from Studenka Village (Glukhsk sub-region of Bykhau region, Mahileu District). The necropolis consists of 107 hemispherical mounds with a rounded shape. The mounds' height ranged from 0.4–2.8m, and 5–16m in diameter. Nearly half of the mounds have marks of disturbances due to different extents, as for example vandal digging or the exploitation of the road that crosses the necropolis.

Mound 96 is located in the south-eastern part of the necropolis. Its height is 1.24 m, length along the north-south line 8.09 m, along the east-west – 6.31 m. The edge width is 0.7 m. The mound is hemispherical in shape being elongated along the north-south line. The mound's body consists of yellow sand with ash and coal increments; the lower ashbin was found 1.15 m from the top of the mound and had a thickness of 2-9 cm.

The mound contained the inhumation burial at the level of the lower ashbin. Although the skeleton was disturbed by the root system of the trees, it was possible to see that the skull was at the east end of the burial, the vertebrae and ribs in the centre, and the leg bones in western part indicating that the body was oriented with the head towards the east. Seventeen fragments of the ceramic pot were found on both sides of the skull. Analysis of the ashbin structure allows the reconstruction of the funeral rite: The body was inhumed at the horizon level; before internment, the burial place was ritually cleansed by fire, the dead body with head oriented eastward was placed in the centre of the place; a mound of 60-65 cm was created above the dead body; at this level the funeral feast was performed and the mound was increased to its final height.

## 1.7 Edix Hill (Barrington A), Cambridgeshire, England (BAEH89/90/91)

*Sarah Inskip*

The cemetery at Edix Hill, also known as Barrington A and distinct from Barrington B, dates from the 5th to 7th century CE. It lies approximately 8 miles south-west of the modern city of Cambridge. Excavations have taken place there since the nineteenth century, but the individual assessed here comes from rescue excavations undertaken by the Cambridgeshire County Council from 1989-1991. In these excavations 115 graves were excavated containing the remains of at least 149 individuals, however, it is thought that over 300 graves could have been originally present. It is archaeologically dated from stratigraphy and grave good typologies supplemented by later radiocarbon dating [64].

The individual sampled was a young middle adult (25-36 years old) female (sk42b) from grave 18B. Sex was confirmed by genetic testing. She was the earliest of three burials in grave17/18, but lay largely undisturbed (Malim and Hines 1998:52). This particular individual is noteworthy for her burial. She is one of two bed burials at the site (the other being Grave 60) and just a handful of bed burials from Western Europe. The burial practice is thought to be reserved for those of higher status. She was buried with multiple grave goods including a glass bead, silver ring (necklace), a key, 2 knives, a bucket, a weaving batten, iron brackets and rod (probably from a wooden box), a comb, a spindle whorl, copper possibly from a pendant, a fossil sea urchin, a sheep astragalus and piece of glass. She was supine extended with her right arm extended and the left flexed over the abdomen. Originally identified as having Hansen's Disease by Corinne Duhig (Malim and Hines 1998), the woman had extensive remodelling to the nasal aperture and loss of the nasal spine. She has periostitis on the tibiae, fibulae and first metatarsal. There was little obvious change to the hands and feet, although it is possible that there was some loss of bone density. One left

manual digit showed evidence for volar grooving. The positive sample was obtained from the upper right canine tooth.

### 1.8 Church End, Cherry Hinton, Cambridgeshire, England (HAT358/1).

*Craig Cessford and Sarah Inskip*

The cemetery at Church End dates from the 10$^{th}$ to the 12$^{th}$ century and lies approximately 6 km southeast of the modern city of Cambridge [65]. It was excavated by the Hertfordshire Archaeological Trust (now Archaeological Solutions) and is situated on land at 69-115 Church End, Cherry Hinton. In the late 9$^{th}$–mid 10$^{th}$ century a large thengly (aristocratic) or manorial centre was established. Associated with this was a timber chapel/church and graveyard which was in use between the 10$^{th}$ and 12$^{th}$ centuries. Only part of the cemetery was investigated, but over 670 graves were excavated and including disarticulated remains c. 980 individuals were identified. The graves were mostly East-West aligned simple earth cut graves. Most individuals were buried in extended supine. Very few grave goods were recovered. As part of the 'After the Plague' project, two individuals were identified as having Hansen's Disease based on skeletal lesions. The burials were typical for the cemetery (West-East extended).

One was an adolescent male (sk2012) who was between 13-17 years in grave 1. Sex was confirmed genetically. He had porosity on the medial surface of the frontal process of the maxilla. There is some evidence of resorption on the frontal process and remodelling of the nasal aperture. There is woven new bone in maxillary sinuses and nasal aperture. Woven new bone is present on the periosteal surfaces of the metatarsals, pedal phalanges, the distal fibulae and tibiae. The positive sample was obtained from an upper right canine.

The second individual (sk 2529) was a young middle (25-36 year old) female in grave 155. Sex was confirmed by genetic testing. She had extensive resorption of the anterior nasal

spine, rounding of nasal aperture and destructive changes penetrating into the left maxillary sinus with porosity and lamellar new bone growth (NBG) in the maxillary sinus. Woven bone was present on the periosteal surfaces of the distal humeri, proximal ulnae, distal left radius, distal tibiae and distal fibulae (resulting in thickened appearance of distal tibiae). It was also observable on the right third metacarpal, the right calcaneus, both first metatarsals and hallucial phalanges. There was no clear concentric remodelling of the phalanges, but these elements are poorly preserved. The positive sample came from an upper right canine.

## 1.9 The Hospital of St John the Evangelist, Cambridge, Cambridgeshire, England (JDS10)

*Craig Cessford and Sarah Inskip*

The individual assessed here, genetically sexed as female, was from disarticulated material associated with the Hospital. The hospital was founded in the town of Cambridge at the very end of the 12th century by the townsfolk, with burial rights acquired in the early 13th century, and was in use until the early 16th century when it was dissolved to create St John’s College, Cambridge. The hospital was principally founded for the care (in a social and spiritual sense, rather than medically) for the ‘poor and infirm’ and for the ‘maintenance of poor scholars and other sick people’; with pregnant women, ‘lepers’, the wounded, ‘cripples’ and the insane all specifically excluded. The cemetery of the hospital was located below the Old Divinity School on St John’s Street. Over 400 complete or partial articulated skeletons were recovered by the Cambridge Archaeological Unit in 2010/2011 [66]. The woman is solely represented by her skull, which does not show any distinct lesions associated with Hansen’s Disease, although the preservation precludes detailed analysis. The positive sample came from an upper left first molar. Radiocarbon dating of stratigraphically associated material indicates that this individual died during the 13th century. The finding of this individual in the

hospital cemetery raises interesting questions since individuals identified with ‘leprosy’ (*leprosis*) are specifically named as being excluded. It is possible that this woman did not manifest typical or extreme external lesions and was in the hospital for other reasons, or for some reason, perhaps relating to status or social relationships, was able to get into the hospital despite her condition. It is also possible that if the woman only developed identifiable signs after she was admitted to the hospital she might have been allowed to remain.

### 1.10 The Hospital of Sant Llàtzer or Santa Margarida, Barcelona, Spain

*Núria Montes Salas*

The Hospital of Sant Llàtzer or Santa Margarida (Saint Lazarus or Saint Margaret), also known as *hospital dels Messells* (the hospital of the ill), was probably founded in the 11th century whose purpose was to shelter all those who suffered from leprosy in Barcelona [67]. The first documents related to the hospital that are preserved are from the end of the 12th century, although its founding date may be earlier [67].

The hospital was established on the edge of the city of Barcelona at the intersection of two main paths that led directly to the city gates (El Portal de la Boqueria and Porta Ferrissa). The Hospital of Colom, founded in 1219, and the Hospital d’en Vilar, from 1311, were also located along the same path. In the 14th century, a third wall was built, and these hospitals were enclosed inside.
According to 12th and 13th century documentary sources, the Hospital of Sant Llàtzer might have sheltered both poor people and people suffering from leprosy simultaneously [67]. However, in the 14th century, once the health assistance network of Barcelona became more complex, only people considered as ‘lepers’ would have been accepted in the hospital [67]. In

1401, the six hospitals of Barcelona merged into one General Hospital, l'Hospital de la Santa Creu, although the *leprosarium* remained at the same place until 1906, when it was moved to a new location outside the city [67].

The account books for 1379 to 1395 have been preserved, which include detailed information about daily life at the hospital, the diet and the origin of the patients [67]. The ill from the city of Barcelona and its surroundings were supposed to have priority for being accepted into the hospital. However, according to the account books, at the end of the $14^{th}$ century many of the patients were foreigners [67]. Moreover, it seems that travelling and pilgrimage were usual among them [67].

Between 2007 and 2009, archaeological works in the surrounding area of the Romanesque church of Sant Llàtzer located part of the cemetery and buildings of the *leprosarium* [68]. The archaeological remains were discovered during the restoration tasks of several buildings of Carme Street and Hospital Street and hence the graves that were located outside the working area remained unexcavated. The excavation was promoted by the city council and the construction company Teyco SL and it was carried out by the archaeological company Atics SL. The remains recovered were housed in the Barcelona History Museum (MUHBA).

The cemetery of the hospital of Sant Llàtzer is the only one directly related to a *leprosarium* that has been excavated in Spain. The cemetery was probably used between the $11^{th}$ and the $18^{th}$ centuries CE. A total of 79 skeletons were recovered from the site during the excavations, corresponding to different chronological periods: $11^{th}$-$13^{th}$ centuries (14 individuals), late $13^{th}$-$14^{th}$ centuries (50 individuals), $15^{th}$-$16^{th}$ centuries (5 individuals) and $17^{th}$-$18^{th}$ centuries (10 individuals). The burials were dated according to associated pottery [68]. Generally, all burials were simple oval shaped structures excavated directly into the soil, except for a single tomb built with flat stones which dated to the $11^{th}$ – early $13^{th}$ century [68]. Some collective burials,

all of them corresponding to the 14th century, were also located. These burials had different levels and were also oval shaped.

Between 1989 and 1991, another extensive archaeological intervention was carried out in the interior of the chapel and several burials corresponding to the 15th – 16th centuries and the 18th century were located [69]. A previous study of the human remains recovered from this excavation concluded that none of the skeletons showed lesions related to leprosy and hence these graves may be related to monks, nuns and wealthy families [69]. Moreover, some burials from the 12th century –early 13th century were also located under the Chapel of Saint Sepulchre, which may correspond to clergymen or workers of the hospital [69].

A total of 35 samples from 18 human skeletons recovered from the cemetery of Sant Llàtzer were used for DNA extraction (Additional file 1: Table S1). The sex of the skeletons was estimated following the standard methods based on the morphology of the cranium and the pelvis [70, 71]. Age at death was estimated from the changes in the auricular surface of the ilium [72] and the pubic symphysis [73]. Regarding non-adult individuals, age estimations were made on the basis of epiphyseal fusion [74] and dental development [75].

For the palaeopathological analysis, the remains were examined macroscopically under white light in the laboratories of the Autonomous University of Barcelona. For those individuals displaying lesions that could be related to Hansen's Disease [76–79], a tooth and a bone sample from an active lesion were selected for DNA extraction whenever possible. The samples were photographed and, in those cases where the bone sample showed an active lesion, an x-ray and a CT scan were also carried out. The radiological analyses and CT scans were performed at the facilities of the Hospital General de Catalunya (General Hospital of Catalonia) by a specialized technician. The samples were handled at all times with nitrile or latex gloves.

Several of the tombs were cut by modern constructions or by other graves and hence some skeletons were incomplete. However, rhinomaxillary changes could be observed in seven of the individuals selected for DNA extraction. A sample from the maxillary bone was taken for the skeletons UF701_UE7016, UF103_UE49, UF11_UE1069 and UF101_UE43, which showed active lesions. For the skeleton UF21_UE1137, the sample was taken from the ethmoid. The skeletons UF703_UE7027 and UF803_UE8020 also showed rhinomaxillary changes, but taking a sample from the maxillary bone would have been too destructive and therefore a hand bone sample was taken instead.

For the remainder of individuals, most of whom did not have a preserved skull, bones from the hands and feet were selected for DNA analyses. Most of them displayed active lesions in the hand bones and/or the feet, except for the skeletons UF800_UE 8008, UF801_UE8011, UF102_UE46 and UF18_UE1123, where the hands and feet were poorly preserved. In those four cases, hand or foot bones without any evident lesions were selected as samples. The only individuals sampled that did not show evident lesions that could be related to Hansen's Disease were UF102_UE46, UF801_UE8011 and UF100_UE40, which were only partially preserved. Skeleton UF100_UE40 showed periostitis on the internal side of the second, third and fourth left ribs as well as on the proximal end of the diaphysis of the left tibia. Moreover, an osteolytic lesion could be observed in the medial phalanx of the 4$^{th}$ ray of their right hand. In this case, a sample of the proximal end of the second left rib was taken in order to test for an infection by *Mycobacterium tuberculosis.*

### 1.11 Blokhuizen, The Netherlands.

*Sarah Inskip*

Archaeological skeletal material from Blokhuizen, located in North Holland, can be broadly dated from the 10$^{th}$ to the 12$^{th}$ century and may relate to a village known as Geddingmore [80]. One hundred and thirty individuals were excavated by the Archeologisch Werkgemeenschap voor Nederlands in 1983 and at the time of research 119 of these were stored at the Laboratory

for Osteoarchaeology and Funerary Archaeology at the University of Leiden. No articulated individuals had evidence for Hansen's Disease, however one disarticulated adult (over 14 years) metatarsal displayed concentric remodelling often associated with the disease. Due to the scarcity of Hansen's Disease cases for the country, in fact there are no published cases at all for the region [1], it was decided that the metatarsal should be sampled and then radiocarbon dated if proving positive. Unfortunately, we were unable to obtain *M.leprae* DNA from the bone.

### 1.12 Santarém, Portugal

*Vitória Duarte, Vitor M. J. Matos, Ana Maria Silva*

Between 2007 and 2008 an archaeological campaign took place in the context of the construction of several residential buildings (Villa Rosa Palace, Avenida 5 de Outubro n. 5-8) in the historical centre of Santarém, a city located in the central region of Portugal. A total of 137 burials and 22 ossuaries were exhumed. Among these, 44 primary burials and 7 ossuaries are possibly associated with the Late Roman and Visigothic periods. One of the individuals (skeleton 2385), a young adult male dated from the $3^{rd}$-$4^{th}$ centuries CE (172-383 cal CE; Beta-524726), found in *decubitus dorsalis* (SW-NE orientation), was diagnosed as a possible case of leprosy. Although this diagnosis is uncertain, it was based on the presence of several post-cranial lesions, which among others included hand and feet proliferative and destructive lesions, such as acroosteolysis in two proximal phalanges of the right foot. The poor preservation of the rhinomaxillary area precluded the observations of eventual leprosy related bone changes in this region [81]. Eight bone samples from skeleton 2385 (S2385 / VRP2385) were collected and analyzed under the scope of this work (Additional file 1: Table S1).

## 1.13 Beja, Portugal

*Nathalie Antunes-Ferreira, Vitor M. J. Matos, Ana Luisa Santos*

The Santo André hermitage (*ermida*) in Beja, south Portugal, was probably founded during the 12$^{th}$ century CE. Documentary sources also indicated the existence of a leprosarium (*gafaria*) between the 14$^{th}$ to 16$^{th}$ centuries, in the surrounding area of this hermitage [82]. The leprosarium necropolis, dated from the Medieval to Early Modern periods, was found during the archaeological excavation that took place in 2003 during hermitage rehabilitation works [35, 83]. The ten graves associated with the leprosarium necropolis were uncovered in survey number 6 (area of 18.6 $m^2$). The graves were oval pits opened on the ground without any delimitation structure. The seven individuals exhumed were found according to Christian canons, in a supine position and with a NE–SW orientation, aligned with the hermitage wall [83]). Three additional skeletons remained in place because they were outside the rehabilitation working area [83]. The first radiocarbon dating (skeleton 3; Beja_3) performed by Oxford University “failed due to very low yield” [35]. The second attempt (skeleton 6) revealed a conventional radiocarbon age of 1265-1313 cal CE (Beta-517063). The preservation of the skeletons was affected by constructions in the area and a drainage pipe [83]. Skeleton 4 (Beja_8) was diagnosed as a probable case of leprosy. This individual presented destructive remodeling in the rhinomaxillary region and leprosy related lesions on metacarpals, metatarsals, and hand and foot phalanges. These lesions were bilateral in both hands and feet, and symmetrical in the feet. Skeletons 1, 3 (Beja_3), 6, and 8 (Beja_8) displayed lesions that are considered possibly – but not probably – related to leprosy [35]. The incompleteness of skeletons 5 and 7 due to anthropic taphonomic factors precluded the observation of the areas of interest. Eleven bone samples from three individuals (Beja_3, Beja_4, Beja_8) were collected and analyzed under the scope of this work (Additional file 2: Table S1).

## 1.14 Travanca, Portugal

*Linda Melo, Vitor M.J. Matos, Ana Luisa Santos, Ana Maria Silva*

An archaeological intervention in the parish church of Saint Mamede in Travanca, a village belonging to the Santa Maria da Feira Municipality, Aveiro district, was carried out between 2016 and 2017. Despite the long use of this Christian space, from the Medieval period ($5^{th}$-$15^{th}$ century CE) to the beginning of the 20th century, only Post-Medieval graves preserved human bone remains. A total of 266 primary burials and 47 ossuaries were recovered from the 412 graves excavated. Among these, individual number 403 (complete reference: IPT_17/K_8/9/UE_2855/Skeleton_403), a poorly preserved and very fragmented skeleton belonging to an adult male, presented several leprosy related bony lesions, namely in the rhinomaxillary area and feet (Melo et al., 2021). This skeleton, radiocarbon dated from the $17^{th}$-$19^{th}$ century CE (Beta 514831), was buried in the churchyard, within a wooden coffin and oriented West-East. Forty-seven rosary beads and a cross with a crucified Jesus Christ were found in the abdominal region and close to the left forearm. This individual represents the first probable evidence of leprosy in Northern Portugal and its funerary context, namely being found with a rosary in a regular cemetery (i.e. not associated to a leprosarium) and without evidence of atypical funerary treatment in death, seems to indicate that during this period, in this geographic region, leprosy patients were not stigmatized and segregated as reported for the Medieval period [84] or he managed to hide his illness. One bone sample (IPT_17) was collected and analyzed under the scope of this work (Additional file 1: Table S1).

## 1.15 Dryburn-Bridge, East Lothian, Scotland

*Alison Sheridan, Charlotte A. Roberts, Philippe Busso, Charlotte Avanzi* and *Stewart T. Cole*

This skeleton (Burial 11) was excavated from the site of Dryburn Bridge, near Innerwick in East Lothian, Scotland, with a site date of 2300–2000 cal BC. Two Late Neolithic/Early Bronze Age burial cists were identified. Cist 2 contained two skeletons (10 and 11). One was

disarticulated (11: child aged 6-8 years) and one articulated (10: older adult male). Roberts J. describes Burial 11 as in a fair condition and 40% complete, and showing '*Resorption of the nasal spine and the region around and above the central incisors, remodelling of the bone and widening of the nasal aperture and slight pitting of the palatal surface. There was evidence of slight new bone growth on the inner surfaces of the nasal bones, and when the face was looked at in profile it had a dished appearance around the nose and mouth area*' [85]. All these bone changes confirmed in 2016 by C. A. Roberts as potentially illustrating rhinomaxillary syndrome. However, tuberculosis and treponemal disease (congenital syphilis) should be considered as differential diagnoses. Nevertheless, while *M. tuberculosis* complex DNA was identified by GM Taylor, it could not be replicated [85]. Samples from the individual also have been screened previously for *M. leprae* by PCR (G.M. Taylor, personal communication) but none was found. However, the primers and protocol used then would not have detected the presence of *M. lepromatosis,* which has been shown to cause leprosy in red squirrels in Scotland and elsewhere [50]. Consequently, DNA was again extracted from this skeleton and screened for both *M. leprae* and *M. lepromatosis* but DNA from neither mycobacterium was detected.

## 1.16 Santa Lucia, Spain

*Natasa Sarkic*

The archaeological site of Santa Lucia belongs to the municipality of Aguilafuente, located 35 km NW of Segovia (Spain). There were noticed two main phases of occupation: Roman villa from lower imperial period, 4$^{th}$ century CE and the Visigoth necropolis from the 6$^{th}$-7$^{th}$ centuries CE [86]. The individual that is the object of our study belongs to the posterior phase, to Visigoth necropolis of Santa Lucía. The skeletal remains were discovered during the excavations carried out in the 2018. The grave of this individual, marked as UE 542, disturbed the previous burial, UE 348. Given the pit of the 542 cuts the pit of 348, it is clear that the 542 was buried

posteriorly. It was noted that the individual 542 did not present the same burial position as the rest of the individuals excavated in the necropolis. All the individuals were in supine position with extended lower extremities and arms extended or folding one or both arms across the chest, with E-W orientation (typical Cristian burial). However, 542 was buried in the flexed left lateral decubitus position, in such forced position that he suffered a postmortem dislocation of the right coxofemoral joint. It was an adult male individual, between 25-30 years of age, and his height was 165 ± 1,98 cm according to Pearson [87].

Pathological changes observed in the skull include the disappearance of the anterior nasal spine, rounding and widening of the nasal opening, destructive remodeling or partial reabsorption of the alveolar process of the anterior maxilla without loss of the upper incisors. Periostitis in the form of isolated plaques was noted in all of the long bones and foot. The alterations present in the hands are very striking and significant, especially in the right hand. Some of the proximal phalanges show periostitis and four of the five metacarpals of the right hand have an abnormal thickening in the diaphysis. Some of them also show signs of porotics changes and bone resorption of the distal epiphysis. The first metatarsal of the right foot shows porotic lesions with osteolysis in its distal third and bone resorption of the distal epiphysis. The third metatarsals and fourth show osteolysis in their distal thirds and the fourth in turn shows porosis. The right fifth metatarsal is pen-shaped with bone retraction in the distal epiphysis and a sequestration and cloaca in the proximal epiphysis. The lesions on the left foot are similar, osteolytic processes are documented in the distal epiphyses of most metatarsals. The fifth metatarsal has lost the distal epiphysis as a result of bone retraction and the phalanx proximal hallux is conical in shape, also due to resorption.

### 1.17 Kich Malka, Russia

*Natalia Berezina, Dimitry Korbov*

Kich-Malka catacomb burial ground was created by the Alans, and is located in the Kislovodsk Basin, North Caucasus, Russia. A female skull from the Kich-Malka catacomb burial ground was discovered during the investigation of the destroyed catacomb. At least nine people were buried in the catacomb: two adults and seven children. The rich complex of grave goods allows to date the burial to the border of the 7-8 centuries CE - the first half of the 8th century CE [88].

# Supplementary Note 2: Radiocarbon Dating

*Saskia Pfrengle*

Since our study focuses on the genetic diversity of ancient M. leprae genomes and the link to historical population dynamic events, it is essential to put the samples into the correct archaeological time. Therefore, it is indispensable to perform radiocarbon dating of the samples of which the genome coverage was suitable to include them to estimate the divergence time by BEAST. In total, 14 samples were directly dated (Table 1, Additional file 1: Table S1, Fig. S1).

For the dating, collagen was extracted from bone and tooth samples according to the established protocols applied in the dating laboratories [89–93]. Finally, the age of the samples was determined by measuring the $^{14}C/^{12}C$ ratio using the MICADAS accelerator mass spectrometry (AMS). Since the dating of the samples was performed at four different laboratories, at the Scottish Universities Environmental Research Center in Glasgow (Kirk Hill and JDS097), the Curt-Engelhorn-Zentrum Archaeometry in Mannheim (Bergen, R7456_671, and PAVd'09_I.5), at the Chrono Center of the Queen's University in Belfast (EDI006, BEL024, CHRY023, and CHRY044), and at the Laboratory of Ion Beam Physics at the ETH Zurich (UF_21, UF_25, UF_101, UF_700, UF_703, and UF_803) the $^{14}C$ data were all calibrated using OxCal v4.4.4 [94, 95].

Our dated samples cover the entire medieval period and range between the 6$^{th}$ century AD and the 16$^{th}$ century AD (Additional file 1: Fig. S1), except the sample R7456_671. This sample is historic and too young to perform exact radiocarbon dating. The calibrated age of the sample was estimated to 18$^{th}$ – 20$^{th}$ century (Additional file 1: Fig. S1).

# Supplementary Note 3: Sample Processing and Genome-wide analyses

*Saskia Pfrengle, Judith Neukamm, Martyna Molak, Meriam Guelli, Marcel Keller, Gunnar U. Neumann*

## 3.1 Sampling

Tübingen and Zurich: To minimize the risk of potential contamination with modern DNA, the surface of all bone and tooth samples were initially UV irradiated from all sides at least for 30 minutes. For DNA extractions, we applied a well-established guanidine-silica based extraction protocol developed for ancient DNA work [151]. For the DNA extraction, we used 30-120 mg of bone powder. For the DNA-extraction step, positive and negative controls were produced; positive controls to determine whether the DNA was successful or not, negative controls to identify potential contamination. The negative controls were carried along with all laboratory experiments and were also sequenced, the positive control till the first step of library preparation.

Cambridge (PSN550) and Tartu (PSN923, PSN951, PSN441, and BEL024): root portions of teeth were removed with a sterile drill wheel or broken off and briefly brushed to remove surface dirt with full strength household bleach (6% w/v NaOCl) using a disposable toothbrush that was soaked in 6% (w/v) bleach prior to use. They were then soaked in 6% (w/v) bleach for 5 minutes. Samples were rinsed twice with 18.2 MΩcm $H_2O$ and soaked in 70% (v/v) Ethanol for 2 minutes and allowed to dry. Samples were incubated for 72 hours at room temperature in a buffer of 2 ml/100 mg sample weight of 0.5M EDTA Buffer pH 8.0 (Fluka) and 50 µl/100 mg sample weight of Proteinase K 10 mg/ml (Roche). Extracts were concentrated to 250 µl using Amplicon Ultra-15 concentrators with a 30 kDa filter (Millipore) and purified according to manufacturer’s instructions using buffers from the Minelute$^{TM}$ PCR

Purification Kit (Qiagen) with the following changes: 1) the use of High-Volume spin columns (Roche); 2) 10X PB buffer instead of 5X; and 3) samples incubated with EB buffer (Qiagen) at 37°C for 10 minutes prior to elution in 100 µl or 50 µl (BEL024) EB buffer. Only one extraction was performed per sample for screening and 30 µl or 50 µl (BEL024) used for libraries.

## 3.2 Library Preparation

### 3.2.1 Double-stranded DNA Libraries

Tübingen and Zurich: The double-stranded DNA library preparation is a two step procedure. In the first step 20 µl of the extracted DNA were converted into double-stranded libraries [152]. In the second step, sample-specific barcodes were added to both ends of the DNA libraries [155]. The indexed sequencing libraries were then amplified again with Herculase II Fusion using the following conditions: 1X Herculase II buffer, 0.4 µM IS5 and 0.4 µM IS6 primer [152] Herculase II Fusion DNA polymerase (Agilent Technologies), 0.25 mM dNTPs (100 mM; 25 mM each dNTP), and 0.5 - 4 µl barcoded library as a template in a total reaction volume of 100 µl. The amplification thermal profile was executed as described: initial denaturation for 2 min at 95°C, denaturation for 30 sec at 95 °C, 30 sec annealing at 60 °C, 30 sec elongation at 72 °C for three to 20 cycles following by a final elongation step for 5 min at 72 °C. Afterwards, the amplified DNA was purified by a MinElute purification step and DNA was eluted in 20 µl TET. We measured the concentration of the amplified sequencing libraries either by using Bioanalyzer (Agilent Technologies) and a DNA1000 lab chip from Agilent Technologies or by Tape Station (Roche).

Tartu: The shotgun library for sample BEL024 was produced in the following manner: Library preparation was conducted using a protocol modified from the manufacturer's instructions included in the NEBNext® Library Preparation Kit for 454 (E6070S, New England Biolabs, Ipswich, MA) as detailed in [153]. Libraries were amplified using the following PCR set up:

30µl DNA library, 1X PCR buffer, 2.5 mM MgCl2, 1 mg/ml BSA, 0.2 µM inPE1.0, 0.2 mM dNTP each, 0.1 U/µl HGS Taq Diamond and 0.2 µM indexing primer. Cycling conditions were: 5' at 94°C, followed by 18 cycles of 30 seconds each at 94°C, 60°C, and 68°C, with a final extension of 7 minutes at 72°C. Amplified products were purified using MinElute columns and eluted in 35 µl EB (Qiagen). Three verification steps were implemented to make sure library preparation was successful and to measure the concentration of dsDNA/sequencing libraries – fluorometric quantitation (Qubit, Thermo Fisher Scientific), parallel capillary electrophoresis (Fragment Analyser, Advanced Analytical) and qPCR.

### 3.2.2 UDG-treated DNA Libraries

The damage patterns of ancient DNA potentially cause sequencing artefacts. These artefacts are problematic for genome-wide analyses. To avoid those potential sequencing artefacts, double-stranded UDG-treated DNA libraries were produced for genome-wide analyses of the samples processed in Tübingen and Zurich. Therefore, 30 µl of the extracted DNA was initially treated with UDG [96]. The indexed double-stranded DNA library preparation and the amplification steps were performed according to the well-established protocols [152, 155].

The library of PSN550 was prepared at the MPI-SHH Jena (Germany) following [154] based on the original UDG protocol by [96]. Two libraries from 25 µl DNA extract each were prepared and pooled after indexing.

The samples PSN923, PSN951, and PSN441, prepared at UTIG Tartu, follows the UDG treatment step according to [154] and continues with Adapter ligation as described for the non-UDG library of BEL024. 30 µl of extract were used as template DNA for the initial USER treatment reaction.

## 3.3 Enrichment strategies

Most of the samples were enriched for the human mitochondrial genome, for three specific leprosy genes, and at least for the complete *M. leprae* genome. The enrichment for the human mitochondrial genome and the three specific leprosy genes were performed by applying an in-solution capture method [156, 157]. Libraries potentially suitable for a whole-genome analysis were enriched for the complete leprosy genome either by an array capture technique [158] or by a myBaits in-solution capture procedure (Arbor Biosciences).

### 3.3.1 Human mitochondrial capture

Selfmade DNA baits [156] covering the complete human mitochondrial (mt) DNA sequence were used to enrich the DNA libraries for the human mt genome's libraries by an in-solution capture approach [157].

### 3.3.2 Mycobacterium leprae gene screening

For screening the samples for *M. leprae* DNA, the libraries were enriched for three specific *M. leprae* genes [44, 45]. These genes are the gyrA gene, specific for all Mycobacteria, and the proS and RLEP genes, typically for *M. leprae* [44]. For the enrichment procedure [157], DNA baits were produced [156] covering the genes' located DNA segments on the *Mycobacterium leprae* genome.

### 2.3.3 *Mycobacterium leprae* genome-wide enrichment

For genome-wide enrichment, UDG treated DNA libraries were used. The enrichment was either performed by an array capture as previously successfully applied and described [44, 45, 158] or by an in-solution capture approach [118] using myBaits Whole Genome Enrichment kit (Arbor Bioscences). Briefly, the array capture approach was performed by two rounds of hybridization of the DNA libraries onto the DNA arrays, containing millions of probes covering the entire *M. leprae* genome [44, 45, 47]. For the in-solution capture, RNA

baits were designed [159], following the manufacturer's protocol, myBaits manual v4 (Arbor Bioscences). For the final amplification, we used Herculase II Fusion polymerase according to the following implementation: 1X Herculase II buffer, 0.4 µM IS5, and 0.4 µM IS6 primer [152], Herculase II Fusion DNA polymerase (Agilent Technologies), 0.25 mM dNTPs (100 mM; 25 mM each dNTP), and 5 µl enriched libraries as a template in a total reaction volume of 100 µl. The thermal amplification profile was executed as described: initial denaturation for 2 min at 95°C, denaturation for 30 sec at 95°C, 30 sec annealing at 60°C, 30 sec elongation at 72°C for 14 cycles following by a final elongation step for 5 min at 72°C. Finally, the amplified DNA was purified by a MinElute purification step and eluted in 20 µl TET.

Samples PSN923, PSN951, PSN441 and BEL024 were processed at the University of Tartu (Estonia) with a different in-solution custom myBaits target enrichment kit from Arbor Biosciences (4X tiling, 70 bp, 19 bp spacing). UDG treated libraries were captured for all samples but BEL024, which was not UDG treated. Target enrichment was performed (individual reactions) following the manufacturer's instructions (myBaits manual v4) in one round of capture with the following exception: half-reactions of baits were used for all samples. We performed a second round of capture for sample PSN951. All samples were amplified using Kapa Hifi Hotstart ReadyMix (2X), and all reactions were purified using Qiagen MinElute columns with a two-step elution and a final elution volume of 30 µl.

## 3.4 DNA sequencing

Sequencing was performed either at the Max Planck Institute for Science of Human History in Jena, at the Functional Genomic Center Zurich in Zurich or at the Institute of Genomics Core Facility at the University of Tartu (UTIG). At the Max Planck Institute for Science of Human History, the sequencing was performed on an Illumina HiSeq4000 platform. Both paired-end and single-end sequencing procedures were applied. Single-end sequencing was executed using 1*75+8+8 cycles, paired-end using 2*50+8+8 cycles. Sequencing at the

Functional Genomic Center Zurich was performed applying a paired-end sequencing approach using either 2*75+8+8 cycles or 2*150+8+8 cycles. The libraries were sequenced on an Illumina NextSeq500 platform or Illumina HiSeq4000 or HiSeq2500 platforms. Samples sequenced at the UTIG were either single-end sequenced on an Illumina NextSeq500 executed applying 1*75+8+8 cycles for an initial screening. The UDG libraries were sequenced on the same sequencing platform. Paired-end sequencing of these libraries was executed using 2*150*8+8 cycles. All three sequencing centres used Illumina standard kits and protocols for sequencing.

## 3.5 Genome-wide analysis - Read processing, mapping, and variant calling

All libraries enriched for the entire *M. leprae* genome were screened using the EAGER pipeline version 1.92.55 [97]. In brief, the quality of the sequencing reads was inspected with FastQC version 0.11.5 [98], all libraries were adapter trimmed and read pairs were merged using AdapterRemoval version 2.2.1a [99] and subsequently aligned to the *M. leprae* reference genome (TN chromosome, NC_002677.1) using CircularMapper version 1.0 [97] with a minimum quality score of 20, a maximum edit distance of n = 0.01 and seeding disabled (recommended parameters that were tested best for aDNA [100, 101]). Relaxed mapping parameters were chosen to take post-mortem damage into account. Duplicates were removed using MarkDuplicates (https://broadinstitute.github.io/picard), and the mapping was evaluated with QualiMap version 2.2.1 [102]. The ancient origin of the reads was verified using DamageProfiler version 1.0 [103]. If a library tested positive (1-fold coverage > 60%), the sample was processed further.

Therefore, all non-UDG treated, adapter-clipped libraries were trimmed by 2bp to remove bases damaged by postmortem damage. Subsequently, all libraries (UDG and non-UDG

treated) were merged by sample and mapped against the *M. leprae* reference genome as described above, only the maximum edit distance was set to n = 0.2. In addition, the UnifiedGenotyper from the Genome Analysis Toolkit (GATK) version 3.8.0 [104, 105] was used to generate a mapping assembly and SNP calling.

### 3.5.1 Processing of published samples

All published modern and ancient strains [44, 45, 47, 49, 106, 114–121] were mapped against the *M. leprae* reference genome as newly sequenced, trimmed samples described above. For the strains where no sequencing reads were available (TN, Br4923), sequencing reads were simulated using Genome2Reads [160] and mapped identically to the other samples.

### 2.5.2 SNP typing

The genotyping of all 19 newly reconstructed strains was performed using an established method [42]. Briefly, there are 84 informative markers (78 SNPs and six InDels in homopolymeric tracts) used for the classification in 16 SNP subtypes of *M. leprae* [42]: 1A-D, 2E-H, 3I-M, and 4 N-P. For a more straightforward application, the SNP types (SNP type 1–4) and the SNP subtypes (A-N) can be determined using a combination of three and 16 loci, respectively [42]. Deeper resolution in SNP subtyping was also recently published and the corresponding specific markers were also applied in our analysis [46].

### 3.5.3 SNP alignment and SNP Effect analysis

The SNP alignment of all modern and ancient published strains [44, 45, 47, 49, 106, 114–121] and the newly sequenced strains was conducted using a modified version of MultiVCFAnalyzer version 0.85.2 [123] (Issue: https://github.com/alexherbig/MultiVCFAnalyzer/issues/5; Pull request: https://github.com/alexherbig/MultiVCFAnalyzer/pull/6). The reference base was called if the

position was covered by a read at least one/three times and the quality score was at least 30. The base was called a SNP if the quality score was at least 30 and 90% of the mapped reads contained this variant. A SNP was used when it was called in at least one sample. If it was not covered or heterozygous in other samples it was set to 'N' there. In addition, all positions were excluded that occur in known repeat regions and rRNA and the positions covered by the negative control sample SK12 [44]. *M. lepromatosis* was used as an outgroup. The pairwise distance of aligned sequences was calculated using snp-dists [124] by considering only the differences of A, C, G, and T (Additional file 3: Table S5).

To investigate the effects of the unique SNPs in our samples that are shared among the newly reconstructed strains, the VCF files for the samples generated in this study were processed using the genomic variant annotations and functional effect prediction toolbox SnpEff version 4.3t [122] to annotate the variants and determine their functional effects. SnpEff was run using default parameters.

### 3.5.4 Phylogeny

The phylogenetic placement of the newly reconstructed strains was performed based on the SNP alignment. Only positions that are covered by at least 80% of the included genomes were considered (partial deletion).

In addition, three different parameter sets were applied to obtain three SNP alignments differing by size and quality =

(1) Using all strains with a 1-fold coverage of at least 60% of the genome to assess the placement of all newly reconstructed low-coverage genomes UF800, COR_XVIII, and UF8. This results in 197 strains and a SNP alignment of 4199 positions.

(2) Using all strains with a 3-fold coverage of at least 60% of the genome. This results in 192 strains and a SNP alignment of 3549 positions.

(3) Using all strains with a 3-fold coverage of at least 60% of the genome, and all hypermutated strains (85054, Amami, S15, Br14-3, Br2016-15, Zensho-4, Zensho-5, and Zensho-9) excluded. This results in 184 strains and a SNP alignment of 2851 positions.

A maximum parsimony (MP) and maximum likelihood (ML) tree were calculated based on the SNP alignment (1) and (2). The MP analysis was performed using MEGAX [108] and 500 bootstraps. The ML tree was determined with RAxML-NG version 1.0.0 [107] and 100 bootstraps using the following command:

```
raxml-ng --all --msa snpAlignmentIncluding.fasta --model GTR+G --tree pars{10} --
bs-trees 100 --threads 16
```

All trees were visualized with FigTree version 1.4.4 (http://tree.bio.ed.ac.uk/software/figtree/) and rooted based on the placement of *M. lepromatosis* .

### 3.5.5 Estimation of divergence time (BEAST analysis)

The SNP alignment (3; see section 2.5.4) was used for phylogenetic timescale estimation and Bayesian phylogenetic inference. The analysis was performed using BEAST 2.6.3 [110] with Bayesian Model test, relaxed log-normal clock and Bayesian Skyline tree prior. The age of each sample was used for the molecular clock calibration. Ancient samples were assigned uniform priors across the most probable age range (95% calibrated $^{14}C$ age estimate or archaeologically assigned) for the tip-date parameter. Two MCMC chains of 100 million steps were run with every 100th step logged and combined using LogCombiner (part of the BEAST package) with 10% burnin steps discarded. Chain convergence and mixing was

inspected in Tracer v1.7.1 [111]. ESS for all but two BMT parameters (gamma shape and proportion of invariable sites) exceeded 200.

Maximum Clade Credibility tree was chosen using TreeAnnotator (part of the BEAST package) and visualised using FigTree v1.4.4 [109].

### 3.5.6 Temporal signal

The temporal signal in the *M. leprae* data set was tested using the Date Randomization Test [112] to assess the applicability of the sample age information to calibrate the molecular clock. The BEAST inference was repeated 10 times using point sample ages (lower end of the age range was used for the ancient samples) randomly reassigned to samples in the data set (otherwise with settings identical to the main BEAST analysis described in the previous paragraph). The mean substitution rate estimate of 95% Credibility Interval for the main BEAST analysis did not overlap with any of the 10 estimated 95% Credibility Intervals in the randomized tip-date runs (Additional file 1: Fig. S6), which indicates sufficient temporal signal for reliable molecular clock calibration. In addition, the temporal signal was investigated using TempEst [113] resulting in R2=0.32 and a correlation coefficient of 0.56 (Additional file 1: Fig. S7).

## 3.6 Human mitochondrial DNA analyses and molecular sex determination

For the analysis of the mitochondrial DNA, the captured data were analyzed by the EAGER pipeline [97]. Reads were mapped against the human mitochondrial genome (NC_012920.1) using CircularMapper [97] with the following settings: a BWA seed length (-l) of 1000 to effectively turn off seeding, BWA Max # Diff (-n) of 0.01 allowing fewer differences of reads to the reference sequence, and BWA quality filter of 30, to discard reads with a lower mapping quality than 30. Schmutzi Contamination Estimation [161] evaluated contamination rates of the sequenced DNA and jointly called the consensus sequence in FASTA format of the

analyzed samples by converting the bam. For consensus calling, bases with low quality are eliminated by applying a quality filter q=20. Only samples with final contamination below 5%, 1st base damage at the 5'-end of the DNA above or equal to 9%, and coverage above 50 % are used for haplogroup determination using HaploFind [162] and HaploGrep2 [163].

For sex determination, the amount of nuclear DNA is mapped to the sex chromosome and autosomal sequences of the complete human genome hg19. For the mapping, the program BWA [164] is used with a BWA seed length (-l) of 32, BWA Max # Diff (-n) of 0.01, and a BWA quality filter (-q): 20. Sex determination is performed applying phyton script for sex identification [165], as well as following the methodology of the sex identification developed by Skoglund and colleagues [166].

$R_{x,}$ the normalized ratio of the alignments to autosomes and sex chromosome X, is calculated. A 95% confidence interval is established. If the upper bound for $R_x$ is lower than 0.60 the individuals' sex is estimated as male and if the lower bound for $R_x$ is higher than 0.80 individuals are estimated as female. For samples with a value for $R_x$ in between the sexes could not be assigned.

We were able to determine the human mitochondrial haplogroup for six individuals and the genetic sex is estimated for three individuals. The results are represented in Additional file 1: Table S1.

# Supplementary Figures

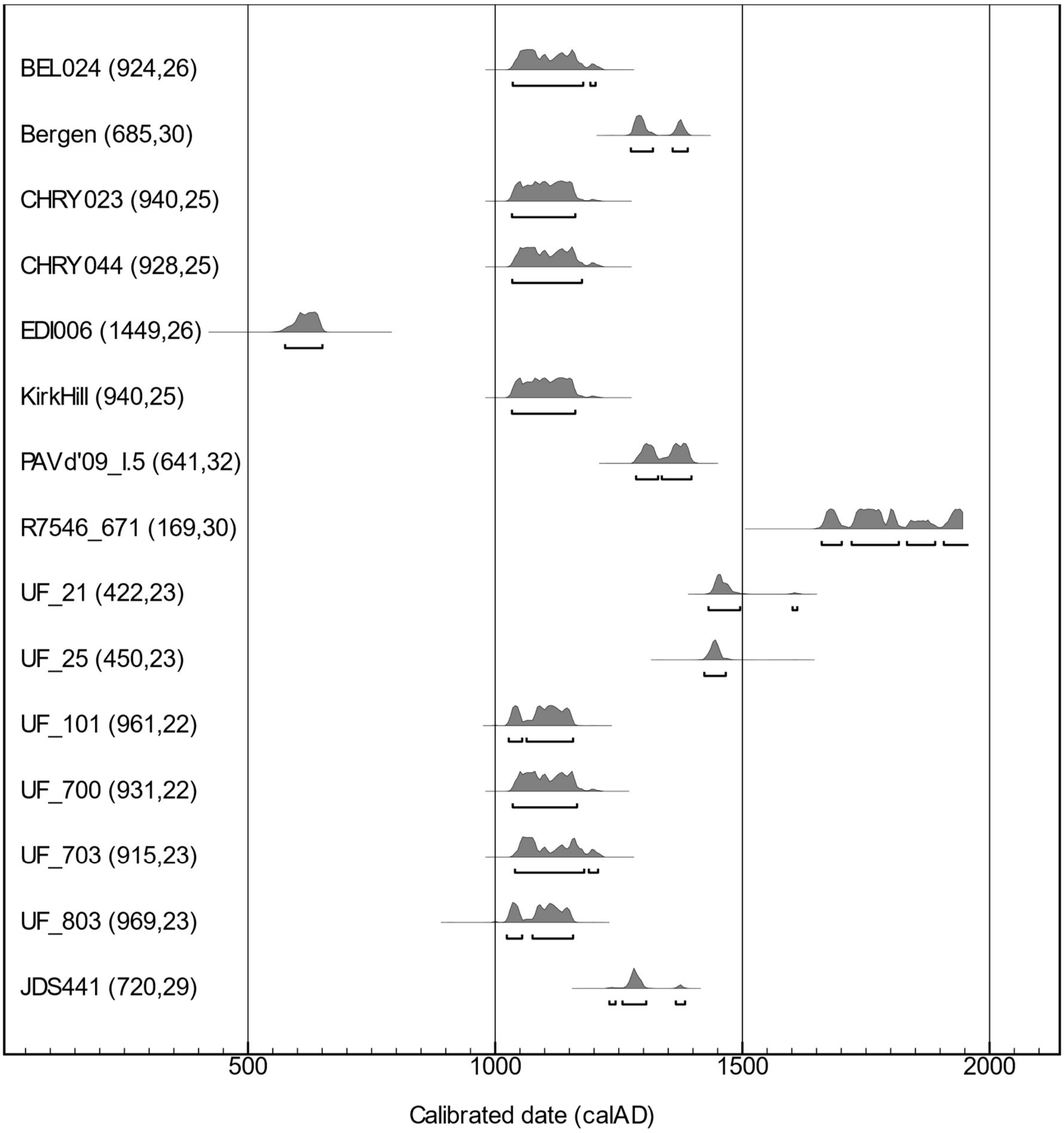


Fig. S1: Radiocarbon dates of the *M. leprae* genomes. Ages are given in cal CE.

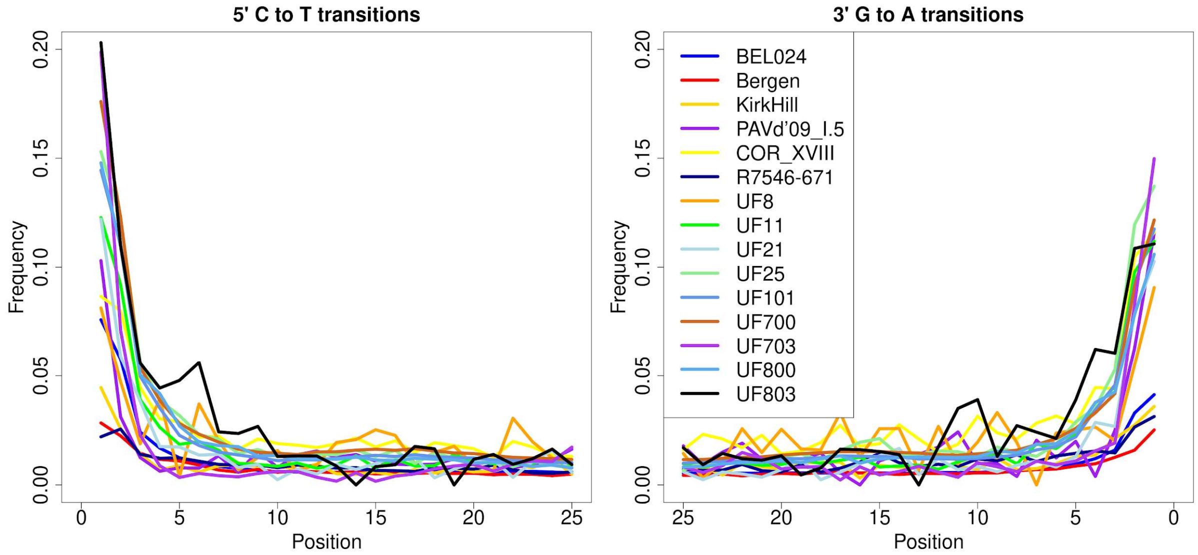


Fig. S2: Damage profiles of all newly reconstructed *M. leprae* strains, where shotgun data were available.

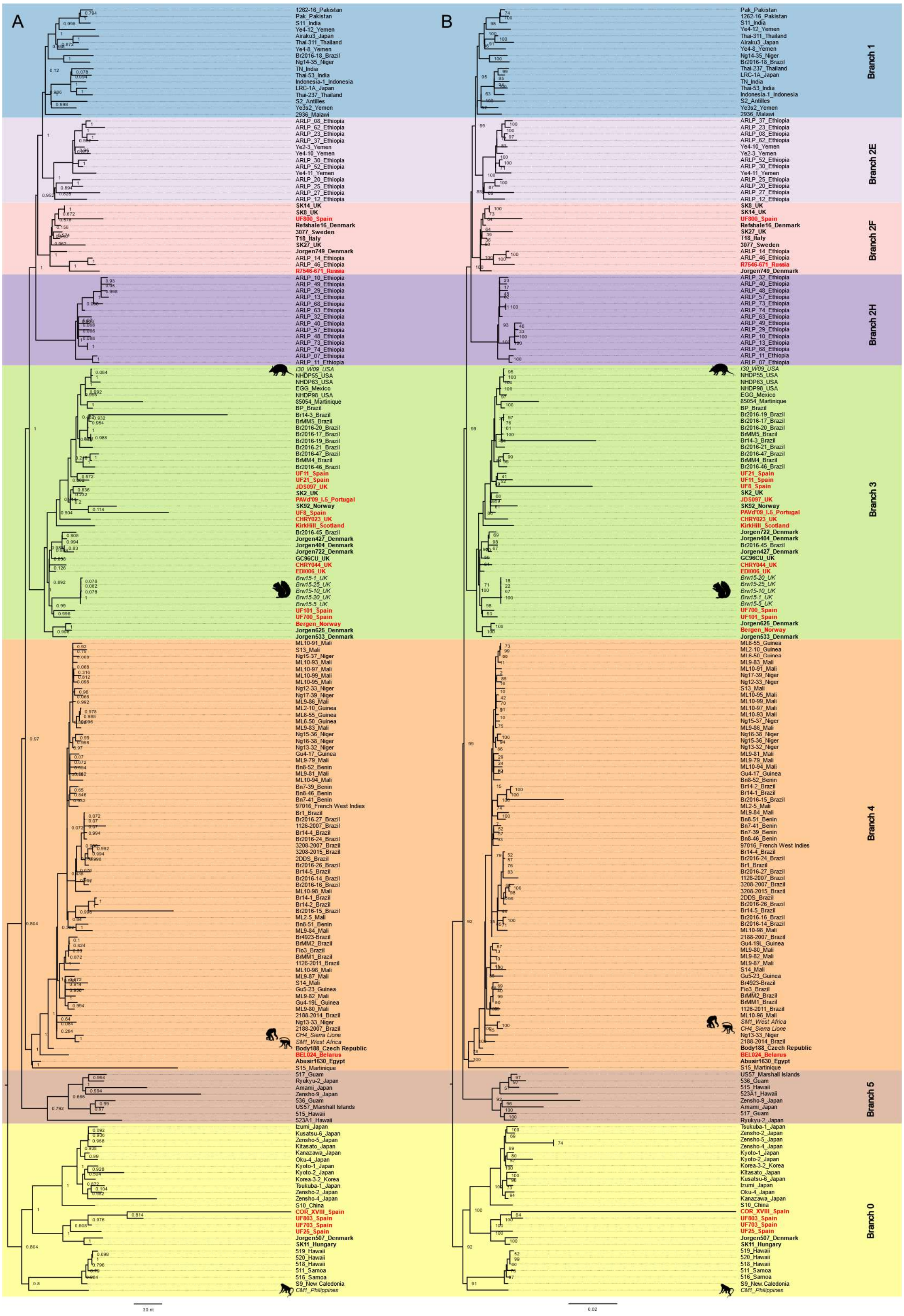


Fig. S3: Uncollapsed (A) Maximum Parsimony and (B) Maximum Likelihood tree using all strains with at least 1-fold coverage at more than 60% of the positions (SNP alignment 1). Ancient strains are in bold, newly added strains highlighted in red. Genomes from animal reservoirs are italic and indicated with the corresponding animal.

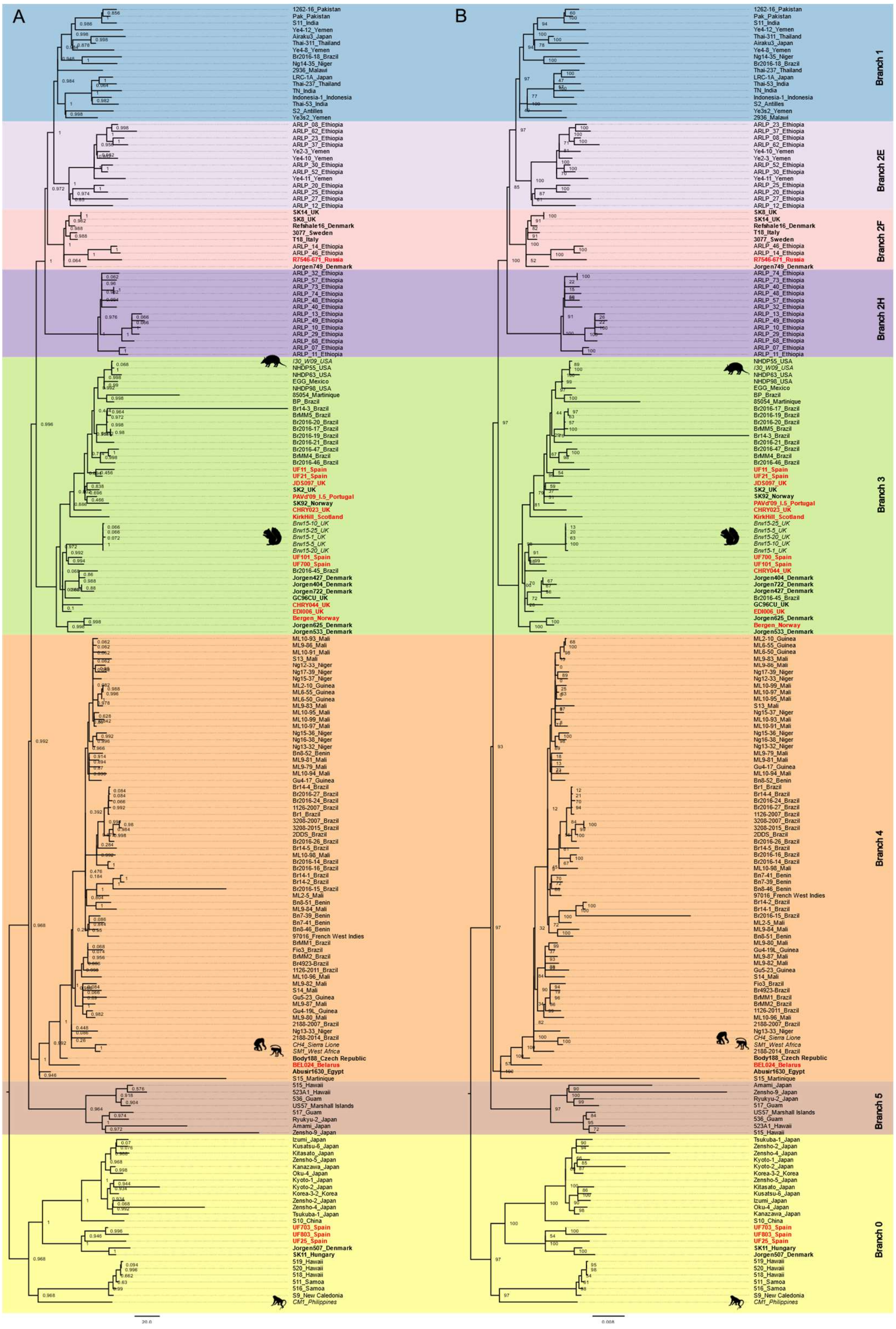


Fig. S4: Uncollapsed (A) Maximum Parsimony and (B) Maximum Likelihood tree using all strains with a 3-fold coverage at more than > 60% of the positions (SNP alignment 2). Ancient strains are in bold, newly added strains highlighted in red. Genomes from animal reservoirs are italic and indicated with the corresponding animal.

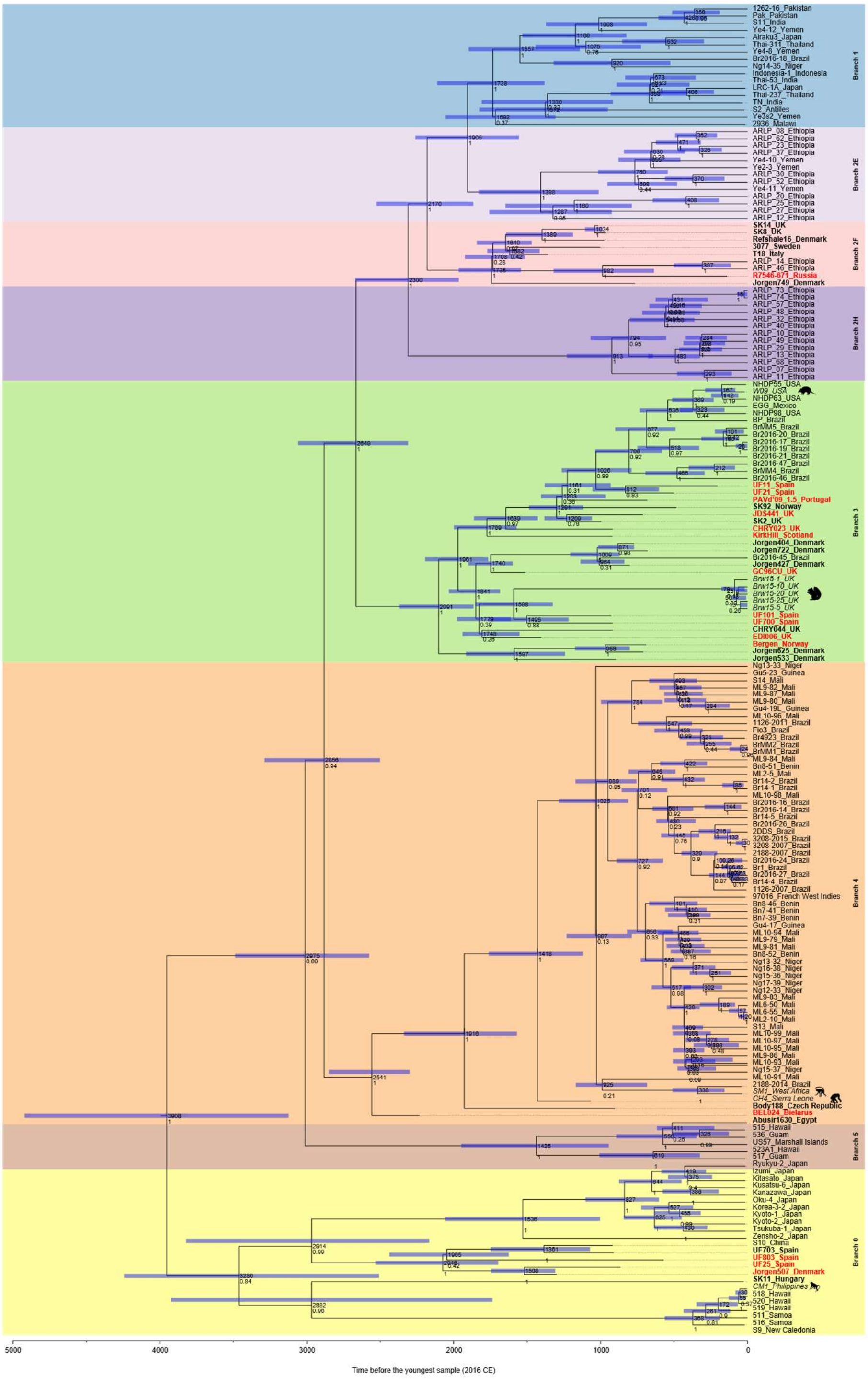


Fig. S5: Uncollapsed Bayesian Maximum Clade Credibility time-aware tree for the leprosy genomes including only genomes with at least 3-fold coverage at least 60% of the genome sites and hypermutated strains excluded (SNP alignment 3). Nodes are labeled with median estimated age (years before 2016 CE, ie. before the youngest sample) and 95% Highest Posterior Density for the age estimate (violet bars) as well as posterior probability estimate.

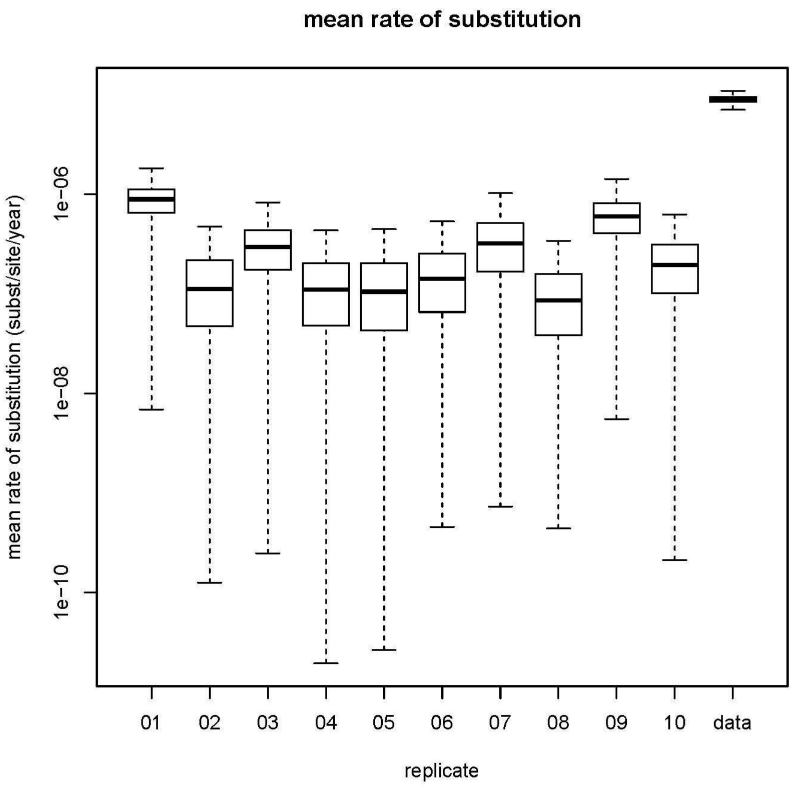


Fig. S6: Date Randomization Test for the *M. leprae* dataset. BEAST analysis was performed for the original data set and ten replicates with randomly reassigned tip calibrations (ages of the samples). The lack of overlap between the timescale parameter estimates (here, the mean rate of nucleotide substitution) indicates a sufficient temporal signal for the molecular clock calibration and time-aware phylogenetic inference.

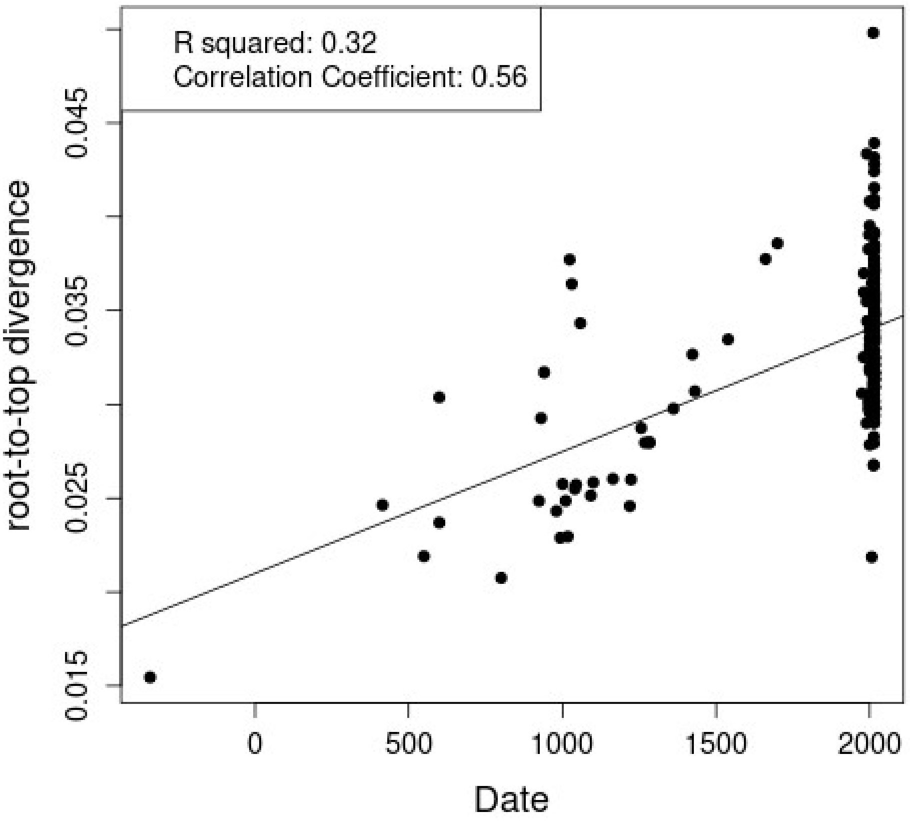


Fig. S7: Result TempEst [113] analysis for the *M. leprae* dataset. The plot visualizes the phylogenetic root-to-tip distance relative to sampling time in years before present with the year 2016 as present.

# Supplementary Tables

Table S1: Sample IDs, archaeological sample name archaeological dates, sample description, age at death, mitochondrial haplogroups, and archaeological and molecular sex of all analysed samples.

| Short ID | Individual | Sample Types Analyzed | Lab ID | Supplier Sample ID | Country | Archaeological Age | 14C age BP | 14C dates (in cal CE) | Dating ID | Age at death | Archaelogical Sex | Molecular Sex (after Mittnik et al., 2016) | Molecular Sex (after Skoglund et al., 2010) | mitochondrial Haplogroup |
|---|---|---|---|---|---|---|---|---|---|---|---|---|---|---|
| Beja3 | Beja_3 | Upper premolar 2 | TU521 | Beja individual no 3 | Portugal | medieval | n.a. | n.a. | n.a | adult | male? | XX (female) | XX (female) | n.a. |
| | Beja_3 | Maxilla | TU522 | | | | | | | | | | | |
| | Beja_3 | Fibula shaft fragment | TU949 | | | | | | | | | | | |
| | Beja_3 | Right rib shaft fragment | TU950 | | | | | | | | | | | |
| Beja4 | Beja_4 | Lower incisor 2 | TU519 | Beja individual no 4 | Portugal | medieval | n.a. | n.a. | n.a | adult | male | XY (male) | consistent with XY but not XX | n.a. |
| | Beja_4 | Navicular right | TU520 | | | | | | | | | | | |
| | Beja_4 | Tibia shaft fragment | TU947 | | | | | | | | | | | |
| | Beja_4 | Unidentified foot fragment | TU948 | | | | | | | | | | | |
| Beja8 | Beja_8 | Tibia | TU523 | Beja individual no 8 | Portugal | medieval | n.a. | n.a. | n.a | adult | male | XY (male) | n.a | n.a. |
| | Beja_8 | Left hand phalanx intermediate | TU951 | | | | | | | | | | | |

| | | | | | | | | | | | | | | |
|---|---|---|---|---|---|---|---|---|---|---|---|---|---|---|
| | Beja_8 | Unidentified foot fragment | TU952 | | | | | | | | | | | |
| Bergen | Bergen | Premolar Tooth | TU594 | Nonneaeter Kloster, Bergen Norway; Era 5 | Norway | medieval | 635-715 | 1268-1388 | MAMS-31414 | n.a. | n.a. | XY (male) | XY (male) | H2a1a |
| Dryburn-Bridge | Dryburn_Bridge | Tooth | TU936 | Dryburn Bridge 1 | UK, Scotland | Early Bronze Age. Scotland | n.a. | n.a. | n.a | 6-7 years | n.a. | XY (male) | XY (male) | n.a. |
| | Dryburn_Bridge | Tooth | TU937 | Dryburn Bridge 2 | | | | | | | | | | |
| | Dryburn_Bridge | Tooth | TU938 | Dryburn Bridge 3 | | | | | | | | | | |
| | Dryburn_Bridge | Septum | TU939 | Dryburn Bridge 4 | | | | | | | | | | |
| IPT17 | IPT_17 | Bone fragment | TU1082 | IPT'17; K-8/9; VE2855;SK403; left fibula | Portugal | 17th c. – early 20th c. | *150-210* | *1642-1911* | *Beta-514831* | adult | male | n.a. | n.a | n.a. |
| KirkHill | Kirk Hill | Temporal bone | TU940 | Kirkhill St. Andrews | UK, Scotland | Early medieval | 915-965 | 1030-1155 | SUERC-91431 | 25-35 years | female | XX (female) | XX (female) | n.a. |
| | Kirk Hill | Skull fragment | TU941 | | | | | | | | | | | |
| | Kirk Hill | Skull fragment | TU942 | | | | | | | | | | | |
| PAVd'09_I.34 | PAVd'09_I.34 | Rib | TU524 | PAVd'09_I.34 | Portugal | $15^{th}$ c. - $17^{th}$ c. | n.a. | n.a. | n.a | adult | male | XX (female) | consistent with XX | n.a. |
| | PAVd'09_I.34 | Manual phalanx | TU525 | | | | | | | | | | | |
| PAVd'09_I.5 | PAVd'09_I.5 | Maxilla | TU526 | PAVd'09_I.5 | Portugal | $15^{th}$ c. - $17^{th}$ c. | 609-673 | 1283-1396 | MAMS-31413 | adult | female | XX (female) | XX (female) | n.a. |
| | PAVd'09_I.5 | Tibia | TU527 | | | | | | | | | | | |
| Blockhuizen | Leiden_Blockhuizen | bone | TU398 | Blokhuizen? Los boc doos leprosy? | Netherlands | $10^{th}$ c. - $12^{th}$ c. | n.a. | n.a. | n.a. | adult | n.a. | n.a. | n.a | n.a. |
| COR_XVIII | COR_XVIII | Fibula shaft fragment | TU1083 | COR_XVIII (Barrejo, Cordiñanes de Valdeón, León) | Spain | $12^{th}$ c. -early $13^{th}$ c. | n.a. | n.a. | n.a. | adult | male | n.a. | n.a | n.a. |
| | COR_XVIII | Tiny bone fragments of the ethmoid | TU1084 | | | | | | | | | | | |
| R7546-671 | Russia_7546-671 | Tooth | TU11 | 7546-671 | Russia | n.a. | 139-199 | 1661-1950 | MAMS-31412 | | female | The sample is | consistent with | n.a. |

| | | | | | | | | | | | | | | |
|---|---|---|---|---|---|---|---|---|---|---|---|---|---|---|
| | | | | | | | | | | | | consistent with XY (male) but not XX (female) | XY but not XX | |
| R7546-695 | Russia_7546-695 | Tooth | TU12 | 7546-695 | Russia | n.a. | n.a. | n.a. | n.a. | n.a. | n.a. | XY (male) | consistent with XY but not XX | F1e3 |
| R-Kich-Malka | Russia_Kich-Malka | Tooth | TU10 | Kich-Malka | Russia | end of 7th – first half of 8th c. | n.a. | n.a. | n.a. | n.a. | n.a. | XX (female) | XX (female) | A12a |
| SLS348B | SantaLucia-Segovia_348B | Tooth | TU1080 | Molar 1; Santa Lucia-Segovia; 348-B | Spain | | n.a. | n.a. | n.a. | n.a. | n.a. | n.a. | n.a | n.a. |
| | SantaLucia-Segovia_348B | Tooth | TU1081 | Molar 2; Santa Lucia-Segovia; 348-B | | | | | | | | | | |
| S2385 | Santarem_2385 | Right upper PM2 | TU1009 | VRP 2385 Santarem | Portugal | Late Roman/ Early medieval | *1730-1790* | *172-383* | *Beta-524726* | adult | male | The sample is consistent with XY (male) but not XX (female) | consistent with XY but not XX | I3a |
| | Santarem_2385 | Metacarpal | TU517 | VRP 2385 Santarem | | | | | | | | | | |
| | Santarem_2385 | Upper premolar right 2 | TU518 | VRP 2385 Santarem | | | | | | | | | | |
| | Santarem_2385 | Right, distal tibia bone fragment | TU943 | VRP 2385 Santarem | | | | | | | | | | |
| | Santarem_2385 | Rib shaft fragment | TU944 | VRP 2385 Santarem | | | | | | | | | | |
| | Santarem_2385 | Left calcaneus bone fragment | TU945 | VRP 2385 Santarem | | | | | | | | | | |
| | Santarem_2385 | Left intermedi | TU946 | VRP 2385 Santarem | | | | | | | | | | |

| | | | | | | | | | | | | | | |
|---|---|---|---|---|---|---|---|---|---|---|---|---|---|---|
| | | ate cuneiform | | | | | | | | | | | | |
| UF100 | UF_100_40 | Proximal end of the second left rib | TU1162 | 045/07 UF 100 UE 40 | Spain | $12^{th}$ c. -early $13^{th}$ c. | n.a. | n.a. | n.a. | 16-17 years | male | n.a. | n.a | n.a. |
| UF101 | UF_101 | Second left maxillary premolar | ZH1108 | 045/07 UF 101 UE 43 | Spain | $12^{th}$ c. - early $13^{th}$ c. | 939-983 | 1027-1157 | ETH-111400 | 25-30 years | male | n.a. | n.a | n.a. |
| | UF_101 | Left portion of the maxillary bone | ZH1119 | 045/07 UF 101 UE 43 | | | | | | | | | | |
| UF102 | UF_102 | Second right mandibular premolar | ZH1117 | 045/07 UF 102 UE46 | Spain | $12^{th}$ c. - early $13^{th}$ c. | n.a. | n.a. | n.a. | 17-20 years | male | n.a. | n.a | n.a. |
| | UF_102 | Right metacarpal 1 | ZH1118 | 045/07 UF 102 UE46 | | | | | | | | | | |
| UF103 | UF_103_49 | Maxillary bone | TU1163 | 045/07 UF 103 UE 49 | Spain | $12^{th}$ c. -early $13^{th}$ c. | n.a. | n.a. | n.a. | 27-35 years | male | n.a. | n.a | n.a. |
| | UF_103_49 | Right mandibular canine | TU1164 | 045/07 UF 103 UE 49 | | | | | | | | | | |
| UF104 | UF_104_56 | Proximal phalanx 1 of the left hand | TU1165 | 045/07 UF 104 UE 56 | Spain | $12^{th}$ c. -early $13^{th}$ c. | n.a. | n.a. | n.a. | n.a. | female | n.a. | n.a | n.a. |
| UF11 | UF_11_69 | Left portion of the maxillary bone | TU1172 | 045/07 UF 11 UE 69 | Spain | $18^{th}$ c. | n.a. | n.a. | n.a | Young adult | female | n.a. | n.a | n.a. |
| | UF_11_69 | Second right mandibular molar | TU1173 | 045/07 UF 11 UE 69 | | | | | | | | | | |
| UF18 | UF_18_1123 | Proximal phalanx 4 of the right foot | TU1174 | 045/07 UF 18 UE 1123 | Spain | $18^{th}$ c. | n.a. | n.a. | n.a | n.a. | n.a. | n.a. | n.a | n.a. |

| | | | | | | | | | | | | | | |
|---|---|---|---|---|---|---|---|---|---|---|---|---|---|---|
| UF21 | UF_21_1137 | First right mandibular molar | TU1170 | 045/07 UF 21 UE 1137 | Spain | 16th c. | 399-445 | 1431-1611 | ETH-107778 | 14-15 years | male | n.a. | n.a | n.a. |
| | UF_21_1137 | Fragment of the ethmoid | TU1171 | 045/07 UF 21 UE 1137 | | | | | | | | | | |
| UF25 | UF_25_1174 | Proximal phalanx 5 of the right hand | TU1166 | 045/07 UF 25 UE 1174 | Spain | end of 13th c. - 14th c. | 427-473 | 1423-1466 | ETH-107776 | 17-20 years | n.a. | n.a. | n.a | n.a. |
| | UF_25_1174 | Second right mandibular molar | TU1167 | 045/07 UF 25 UE 1174 | | | | | | | | | | |
| UF700 | UF_700 | Right metacarpal 3 | ZH1113 | 045/07 UF 700 UE7006 | Spain | 12th c. - early 13th c. | 909-953 | 1035-1165 | ETH-111399 | 17-20 years | female | n.a. | n.a | n.a. |
| | UF_700 | Left metacarpal 2 | ZH1114 | 045/07 UF 700 UE7006 | | | | | | | | | | |
| UF701 | UF_701_7016 | Left portion of the maxillary bone | TU1152 | 045/07 UF 701 UE 7016 | Spain | 12th c. -early 13th c. | n.a. | n.a. | n.a. | n.a. | female | n.a. | n.a | n.a. |
| | UF_701_7016 | First right maxillary molar | TU1153 | 045/07 UF 701 UE 7016 | | | | | | | | | | |
| | UF_701_7016 | Head of the left humerus | TU1154 | 045/07 UF 701 UE 7016 | | | | | | | | | | |
| UF702 | UF_702_7019 | Proximal phalanx 1 of the right hand | TU1155 | 045/07 UF 702 UE 7019 | Spain | 12th c. -early 13th c. | n.a. | n.a. | n.a. | n.a. | male | n.a. | n.a | n.a. |
| | UF_702_7019 | Third left mandibular molar | TU1156 | 045/07 UF 702 UE 7019 | | | | | | | | | | |
| UF703 | UF_703_7027 | Left metacarpal 3 | TU1157 | 045/07 UF 703 UE 7027 | Spain | 12th c. -early 13th c. | 892-938 | 1040-1208 | ETH-107775 | 25-35 years | female | n.a. | n.a | n.a. |
| | UF_703_7027 | First right mandibular premolar | TU1158 | 045/07 UF 703 UE 7027 | | | | | | | | | | |

| | | | | | | | | | | | | | | |
|---|---|---|---|---|---|---|---|---|---|---|---|---|---|---|
| UF8 | UF_8_1054 | Right metatarsus 4 | TU1168 | 045/07 UF 8 UE 1054 | Spain | 16th c. | n.a. | n.a. | n.a | n.a. | female | n.a. | n.a | n.a. |
| | UF_8_1054 | Second left mandibular molar | TU1169 | 045/07 UF 8 UE 1054 | | | | | | | | | | |
| UF800 | UF_800 | Right metacarpal 1 | ZH1111 | 045/07 UF 800 UE 8008 | Spain | 12th c. - early 13th c. | n.a. | n.a. | n.a | 12-14 years | female | n.a. | n.a | n.a. |
| | UF_800 | Right metacarpal 3 | ZH1112 | 045/07 UF 800 UE 8008 | | | | | | | | | | |
| UF801 | UF_801 | Left metatarsus 5 | ZH1109 | 045/07 UF 801 UE 8011 | Spain | 12th c. - early 13th c. | n.a. | n.a. | n.a. | 16-20 years | n.a. | n.a. | n.a | n.a. |
| | UF_801 | Proximal phalanx 4 of the left hand | ZH1110 | 045/07 UF 801 UE 8011 | | | | | | | | | | |
| UF802 | UF_802 | Proximal phalanx 2 of the right hand | ZH1115 | 045/07 UF 802 UE 8014 | Spain | 12th c. - early 13th c. | n.a. | n.a. | n.a. | 35-45 years | female | n.a. | n.a | n.a. |
| | UF_802 | Left metatarsus 5 | ZH1116 | 045/07 UF 802 UE 8014 | | | | | | | | | | |
| UF803 | UF_803_8020 | Proximal phalanx 2 of the right hand | TU1159 | 045/07 UF 803 UE 8020 | Spain | 12th c. -early 13th c. | 946-992 | 1023-1157 | ETH-107777 | 25-30 years | female | n.a. | n.a | n.a. |
| | UF_803_8020 | Third left mandibular molar | TU1160 | 045/07 UF 803 UE 8020 | | | | | | | | | | |
| | UF_803_8020 | Medial phalanx 5 of the left hand | TU1161 | 045/07 UF 803 UE 8020 | | | | | | | | | | |
| BEL024 | 24 | bone | BEL024 / II24 | 24 | Belarus | 10th c. - 12th c. | 898-950 | 135-1203 | UBA-44327 | 25-30 years | male | n.a. | n.a | n.a. |
| PSN932 | sk. 2012 | bone | CHRY023 | PSN 932 | UK, England | 10th c. - 12th c. | 915-965 | 1034-1162 | UBA-44325 | n.a. | n.a. | n.a. | n.a | n.a. |
| PSN951 | sk. 2529 | Upper right canine tooth | CHRY044 | PSN 951 | UK, England | 10th c. - 12th c. | 903-953 | 1034-1162 | UBA-44326 | n.a. | n.a. | n.a. | n.a | n.a. |

| | | | | | | | | | | | | | | |
|---|---|---|---|---|---|---|---|---|---|---|---|---|---|---|
| PSN550 | sk42b | Upper right canine tooth | EDI006 | PSN 550 | UK, England | $5^{th}$ c. - $7^{th}$ c. (550-650 CE) | 1423-1475 | 575-650 | UBA-44321 | 25-36 years | female | female | female | n.a. |
| PSN441 | JDS10 | Upper left first molar | JDS097 | PSN 441 | UK, England | $12^{th}$ c. -early $16^{th}$ c. | 691-749 | 1231–1384 | SUERC-71631 | n.a. | n.a. | female | female | n.a. |

Table S2: Eager Report of the analysed samples (nonUDG - and UDG-treated merged and trimmed).

| Sample Name | # reads after C&M Prior mapping | # mapped reads Prior RMDup | # of Duplicates Removed | Mapped Reads After RMDup | Endog. DNA (%) | Cluster Factor | Mean Coverage | std. dev. Coverage | Coverage >= 1X in % | Coverage >= 3X in % | Coverage >= 5X in % | # SNPs | DMG 1st Base 3' | DMG 2nd Base 3' | DMG 1st Base 5' | DMG 2nd Base 5' | Avg. frag. Length | Med. frag. Length | GC content In % |
|---|---|---|---|---|---|---|---|---|---|---|---|---|---|---|---|---|---|---|---|
| BEL024 | 29078430 | 8230009 | 6564253 | 1665756 | 28.3 | 4.94 | 43.86 | 11.85 | 97.71 | 97.51 | 97.41 | 111 | 0.01 | 0.01 | 0.02 | 0.02 | 86.06 | 74 | 56.87 |
| PSN550 | 17226020 | 1482922 | 488509 | 994413 | 8.61 | 1.49 | 23.71 | 7.59 | 97.64 | 97.43 | 97.27 | 98 | 0 | 0 | 0 | 0 | 77.92 | 76 | 57 |
| UF700 | 18284857 | 5035703 | 4150452 | 885251 | 27.54 | 5.69 | 19.45 | 9.2 | 97.53 | 96.91 | 95.39 | 105 | 0.04 | 0.03 | 0.05 | 0.04 | 71.79 | 68 | 55.43 |
| Bergen | 136380452 | 37218175 | 32134682 | 5083493 | 27.29 | 7.32 | 110.61 | 29.9 | 97.45 | 97.44 | 97.43 | 114 | 0.01 | 0 | 0 | 0 | 71.11 | 76 | 56.58 |
| PAVd'09_I.5 | 195205512 | 68614256 | 63831256 | 4783000 | 35.15 | 14.35 | 96.82 | 23.75 | 97.45 | 97.44 | 97.42 | 115 | 0.02 | 0.01 | 0 | 0 | 66.15 | 74 | 56.72 |
| UF703 | 32430019 | 5269402 | 3996645 | 1272757 | 16.25 | 4.14 | 26.94 | 18.03 | 97.44 | 96.19 | 93.73 | 92 | 0.01 | 0 | 0 | 0 | 69.17 | 67 | 54.42 |
| UF25 | 33323580 | 5048917 | 3481969 | 1566948 | 15.15 | 3.22 | 33.09 | 24.92 | 97.4 | 95.73 | 92.92 | 167 | 0 | 0 | 0 | 0 | 69.01 | 66 | 54.02 |
| UF101 | 31998474 | 3959782 | 2971600 | 988182 | 12.38 | 4.01 | 21.28 | 13.77 | 97.39 | 95.69 | 92.41 | 99 | 0.04 | 0.03 | 0.05 | 0.04 | 70.37 | 68 | 54.05 |
| R7546-671 | 214398970 | 21378975 | 20459735 | 919240 | 9.97 | 23.26 | 16.51 | 11.79 | 97.16 | 94.96 | 90.16 | 103 | 0 | 0 | 0 | 0 | 58.71 | 59 | 53.14 |
| PSN441 | 13563704 | 5369924 | 4628085 | 741839 | 39.59 | 7.24 | 12.81 | 6.84 | 96.89 | 94.27 | 89.02 | 102 | 0 | 0 | 0 | 0 | 56.44 | 54 | 56.11 |

| | | | | | | | | | | | | | | | | | | |
|---|---|---|---|---|---|---|---|---|---|---|---|---|---|---|---|---|---|---|
| PSN923 | 21321540 | 2870513 | 2530422 | 340091 | 13.46 | 8.44 | 7.01 | 3.66 | 96.53 | 89.75 | 74.59 | 90 | 0 | 0 | 0 | 0 | 67.34 | 65 | 56.6 |
| PSN951 | 15621609 | 14588832 | 13533611 | 1055221 | 93.39 | 13.83 | 18.09 | 11.67 | 96.31 | 92.35 | 87.07 | 90 | 0 | 0 | 0 | 0 | 56.04 | 53 | 55.31 |
| KirkHill | 67922658 | 2275591 | 1911112 | 364479 | 3.35 | 6.24 | 6.86 | 5.64 | 94.85 | 81.01 | 62.36 | 74 | 0 | 0 | 0 | 0 | 61.51 | 58 | 53.63 |
| UF21 | 29619414 | 1660187 | 1451001 | 209186 | 5.61 | 7.94 | 4.11 | 3.26 | 92.14 | 67.7 | 39.31 | 51 | 0.01 | 0 | 0.01 | 0 | 64.21 | 60 | 55.3 |
| UF803 | 90338982 | 1220373 | 945382 | 274991 | 1.35 | 4.44 | 6.18 | 6.18 | 91.01 | 69.77 | 50.67 | 110 | 0 | 0 | 0 | 0 | 73.49 | 74 | 53.73 |
| UF800 | 147138794 | 499762 | 351764 | 147998 | 0.34 | 3.38 | 3.34 | 3.82 | 86.27 | 52.63 | 27.09 | 49 | 0.01 | 0.01 | 0.02 | 0.02 | 73.79 | 72 | 54.52 |
| UF11 | 25123610 | 2323783 | 1976038 | 347745 | 9.25 | 6.68 | 6.71 | 8.08 | 85.67 | 61.81 | 45.56 | 71 | 0.01 | 0 | 0.01 | 0 | 63.05 | 60 | 51.8 |
| UF8 | 29735005 | 784616 | 715814 | 68802 | 2.64 | 11.4 | 1.46 | 2.89 | 67.52 | 18.99 | 3.43 | 75 | 0 | 0 | 0 | 0 | 69.16 | 68 | 55.19 |
| COR_XVIII | 119588632 | 2192831 | 2034738 | 158093 | 1.83 | 13.87 | 2.49 | 4.67 | 67.28 | 32.39 | 17.55 | 154 | 0 | 0 | 0 | 0 | 51.51 | 49 | 52.06 |
| Beja_3 | 81573014 | 711528 | 678407 | 33121 | 0.87 | 21.48 | 0.56 | 3.62 | 29.66 | 2.23 | 0.66 | 276 | 0.02 | 0.01 | 0.01 | 0.01 | 55.36 | 51 | 54.57 |
| Beja_4 | 120564575 | 1786397 | 1761171 | 25226 | 1.48 | 70.82 | 0.38 | 4.57 | 5.87 | 1.72 | 1.23 | 742 | 0.03 | 0.02 | 0.02 | 0.01 | 49.2 | 45 | 59.35 |
| Beja_8 | 121591590 | 715294 | 700941 | 14353 | 0.59 | 49.84 | 0.23 | 3.95 | 2.65 | 0.88 | 0.62 | 266 | 0.02 | 0.02 | 0.01 | 0.01 | 53.08 | 47 | 59.43 |
| UF801 | 45978753 | 5334 | 2879 | 2455 | 0.01 | 2.17 | 0.04 | 1.04 | 1.99 | 0.13 | 0.09 | 41 | 0.02 | 0.01 | 0.02 | 0.02 | 58.68 | 51 | 55.62 |

| | | | | | | | | | | | | | | | | | | |
|---|---|---|---|---|---|---|---|---|---|---|---|---|---|---|---|---|---|---|
| Dryburn_Bridge | 20848910 | 5519 | 3117 | 2402 | 0.03 | 2.3 | 0.03 | 1.11 | 1.07 | 0.1 | 0.08 | 52 | 0.03 | 0.02 | 0.03 | 0.02 | 47.22 | 49 | 56.36 |
| Santarem | 41125295 | 12310 | 9133 | 3177 | 0.03 | 3.88 | 0.05 | 1.65 | 1.02 | 0.15 | 0.12 | 51 | 0.08 | 0.04 | 0.04 | 0.03 | 52.03 | 49 | 56.75 |
| Karganaee | 19725425 | 2167 | 839 | 1328 | 0.01 | 1.63 | 0.02 | 0.98 | 0.26 | 0.1 | 0.07 | 17 | 0.03 | 0.02 | 0.02 | 0.01 | 56.84 | 55 | 57.36 |
| R7546-695 | 55145609 | 10340 | 8961 | 1379 | 0.02 | 7.5 | 0.02 | 0.96 | 0.24 | 0.11 | 0.08 | 21 | 0.03 | 0.02 | 0.02 | 0.02 | 55 | 51 | 56.18 |
| UF102 | 4720599 | 2395 | 2105 | 290 | 0.05 | 8.26 | 0 | 0.2 | 0.24 | 0.03 | 0.01 | 4 | 0.01 | 0 | 0.04 | 0.02 | 53.93 | 46 | 55.67 |
| Russia Kich-Malka | 108444280 | 6760 | 5666 | 1094 | 0.01 | 6.18 | 0.02 | 0.74 | 0.2 | 0.08 | 0.06 | 6 | 0.23 | 0.03 | 0.06 | 0.02 | 49.28 | 46 | 55.73 |
| Russia Sajanskaja | 36055190 | 10270 | 8934 | 1336 | 0.03 | 7.69 | 0.02 | 0.93 | 0.2 | 0.1 | 0.07 | 20 | 0.03 | 0.02 | 0.02 | 0.01 | 53.55 | 50 | 56.17 |
| UF701 | 8430829 | 2501 | 1985 | 516 | 0.03 | 4.85 | 0.01 | 0.41 | 0.17 | 0.05 | 0.04 | 7 | 0.01 | 0.02 | 0.05 | 0.01 | 47.7 | 44 | 56.87 |
| Lagos34 | 11279453 | 2316 | 1196 | 1120 | 0.02 | 2.07 | 0.02 | 0.9 | 0.16 | 0.07 | 0.05 | 34 | 0.42 | 0.03 | 0.02 | 0.01 | 54.36 | 51 | 55.3 |
| UF103 | 5584524 | 2652 | 1541 | 1111 | 0.05 | 2.39 | 0.02 | 0.85 | 0.16 | 0.07 | 0.06 | 48 | 0.02 | 0.02 | 0.02 | 0.02 | 52.79 | 50 | 55.71 |
| UF18 | 2256635 | 1524 | 1422 | 102 | 0.07 | 14.94 | 0 | 0.09 | 0.11 | 0.01 | 0 | 2 | 0 | 0 | 0 | 0.07 | 55.03 | 48 | 56.71 |
| UF702 | 3728426 | 869 | 343 | 526 | 0.02 | 1.65 | 0.01 | 0.37 | 0.11 | 0.05 | 0.04 | 18 | 0.03 | 0.04 | 0.07 | 0.04 | 42.63 | 42 | 56.09 |
| UF100 | 2115386 | 320 | 109 | 211 | 0.02 | 1.52 | 0 | 0.17 | 0.08 | 0.03 | 0.02 | 3 | 0.04 | 0 | 0.05 | 0 | 44.07 | 43 | 56.55 |

| | | | | | | | | | | | | | | | | | | | |
|---|---|---|---|---|---|---|---|---|---|---|---|---|---|---|---|---|---|---|---|
| UF802 | 2670874 | 232 | 59 | 173 | 0.01 | 1.34 | 0 | 0.17 | 0.07 | 0.03 | 0.02 | 3 | 0.1 | 0 | 0.04 | 0.06 | 45.64 | 43 | 56.07 |
| Leiden Blockhuizen | 3041762 | 558 | 380 | 178 | 0.02 | 3.14 | 0 | 0.16 | 0.05 | 0.03 | 0.02 | 4 | 0.05 | 0.02 | 0.02 | 0 | 46.61 | 46 | 55.44 |
| UF104 | 2060458 | 208 | 101 | 107 | 0.01 | 1.94 | 0 | 0.12 | 0.05 | 0.01 | 0.01 | 1 | 0.04 | 0 | 0.04 | 0.04 | 43.19 | 41 | 56.42 |
| SantaLucia Segovia | 237189 | 34 | 5 | 29 | 0.01 | 1.17 | 0 | 0.03 | 0.02 | 0 | 0 | 0 | 0 | 0 | 0 | 0 | 44 | 43 | 56.19 |
| IPT_17 | 109208 | 19 | 10 | 9 | 0.02 | 2.11 | 0 | 0.01 | 0.01 | 0 | 0 | 0 | 0 | 0 | 0 | 0 | 48.56 | 46 | 57.67 |

Table S3: SNP subtyping [42, 45–47, 106]

| | *M. leprae* coordinates (strain TN as reference) | | | | | | | | | | | |
|---|---|---|---|---|---|---|---|---|---|---|---|---|
| | 1642875 | 2935685 | 14676 | 3102778 | 1104232 | 7614 | 1527056 | 2312059 | 711197 | Monot* | Genotype (deeper resolution)** | branch |
| UF800 | T | A | C | C | C | C | G | C | T | 2F | 2F | 2F |
| R7546-671 | T | A | C | C | C | C | G | C | T | 2F | 2F | 2F |
| UF11 | T | C | C | C | G | T | G | C | T | 3I | 3I-1 | 3 |
| UF21 | T | C | C | C | G | T | G | C | T | 3I | 3I-1 | 3 |
| UF8 | T | C | C | C | G | T | G | C | T | 3I | 3I-1 | 3 |
| PAVd'09_I.5 | T | C | C | C | G | T | G | C | T | 3I | 3I-1 | 3 |
| PSN923 | T | C | C | C | G | T | G | C | T | 3I | 3I-1 | 3 |
| KirkHill | T | C | C | C | G | T | G | C | T | 3I | 3I-1 | 3 |
| JDS5097 | T | C | C | C | G | T | G | C | T | 3I | 3I-1 | 3 |
| UF101 | T | C | C | C | G | T | G | C | T | 3I | 3I-1 | 3 |
| UF700 | T | C | C | C | G | T | G | C | T | 3I | 3I-1 | 3 |
| PSN951 | T | C | C | C | G | T | G | C | T | 3I | 3I-1 | 3 |
| Bergen | T | C | C | C | G | T | G | C | T | 3I | 3I-1 | 3 |
| PSN550 | T | C | C | C | G | T | G | C | T | 3I | 3I-1 | 3 |
| BEL024 | T | C | C | C | G | C | G | C | T | 3L | New (3Q) | 4 |
| COR_XVIII | T | C | C | C | G | C | G | G | C | 3K | 3K-0 | 0 |
| UF803 | T | C | C | C | G | C | G | G | C | 3K | 3K-0 | 0 |
| UF703 | T | C | C | C | G | C | G | G | C | 3K | 3K-0 | 0 |
| U25 | T | C | C | C | G | C | G | G | C | 3K | 3K-0 | 0 |

* according to the 16 loci described by Monot et al. [42, 106].

** according to the loci described by Truman *et al.* [106].

Table S4: Result table of the SNP effect analysis (Additional file 2)

Table S5: SNP distance matrix based on alignment 2 (2.5.4 Phylogeny). (Additional file 3)

Table S6: Unique SNPs within the newly reconstructed strains located within genes that are related to virulence factors (according: http://www.mgc.ac.cn/cgi-bin/VFs/compvfs.cgi).

| Gene name | Strain | Position of SNP (TN reference) | Coverage | Protein function associate with virulence factor |
|---|---|---|---|---|
| *leuD* | PSN951 | 2029811 | 29X | Amino acid and purine metabolism / Leucine synthesis |
| *mce1A* | UF800 | 3093139 | 3X | Mammalian cell entry (mce) operons |
| *ml0049* | UF700 | 61431 | 13X | Secretion system / ESX-1 (T7SS) |
| *ml0135* | BEL024 | 184256 | 59X | Cell surface components / PDIM (phthiocerol dimycocerosate) and PGL (phenolic glycolipid) biosynthesis and transport |
| *ml1539* | UF703 | 1856699 | 11X | Secretion system / ESX-5 (T7SS) |
| *ml2534* | UF703 | 3016347 | 8X | Secretion system / ESX-3 (T7SS) |
